# Design and Synthesis of Novel Indole Ethylamine Derivatives as a Lipid Metabolism Regulator Targeting PPARα/CPT1 in AML12 Cells

**DOI:** 10.3390/molecules29010012

**Published:** 2023-12-19

**Authors:** Yu-Chen Liu, Gang Wei, Zhi-Qiang Liao, Fang-Xin Wang, Chunxiao Zong, Jiannan Qiu, Yifei Le, Zhi-Ling Yu, Seo Young Yang, Heng-Shan Wang, Xiao-Bing Dou, Cai-Yi Wang

**Affiliations:** 1 College of Life Science, Zhejiang Chinese Medical University, Hangzhou 310053, China; 17774008010@163.com (Y.-C.L.); z1c10x1@163.com (C.Z.); qjntcm@163.com (J.Q.); lyf19970605@foxmail.com (Y.L.); 2 State Key Laboratory for Chemistry and Molecular Engineering of Medicinal Resources/Key Laboratory for Chemistry and Molecular Engineering of Medicinal Resources (Ministry of Education of China), School of Chemistry and Pharmaceutical Sciences, Collaborative Innovation Center for Guangxi Ethnic Medicine, Guangxi Normal University, Guilin 541004, China; wei18249913020@163.com (G.W.); 15077302639@163.com (Z.-Q.L.); wangfx@mailbox.gxnu.edu.cn (F.-X.W.); whengshan@163.com (H.-S.W.); 3 School of Chinese Medicine, Hong Kong Baptist University, Hong Kong, China; zlyu@hkbu.edu.hk; 4 Department of Biology Education, Teachers College and Institute for Phylogenomics and Evolution, Kyungpook National University, Daegu 41566, Republic of Korea; syy@knu.ac.kr

**Keywords:** NAFLD, PPARα, CPT1, indole ethylamine derivatives

## Abstract

Peroxisome proliferator-activated receptor alpha (PPARα) and carnitine palmitoyltransferase 1 (CPT1) are important targets of lipid metabolism regulation for nonalcoholic fatty liver disease (NAFLD) therapy. In the present study, a set of novel indole ethylamine derivatives (**4**, **5**, **8**, **9**) were designed and synthesized. The target product (compound **9**) can effectively activate PPARα and CPT1a. Consistently, in vitro assays demonstrated its impact on the lipid accumulation of oleic acid (OA)-induced AML12 cells. Compared with AML12 cells treated only with OA, supplementation with 5, 10, and 20 μM of compound **9** reduced the levels of intracellular triglyceride (by 28.07%, 37.55%, and 51.33%) with greater inhibitory activity relative to the commercial PPARα agonist fenofibrate. Moreover, the compound **9** supplementations upregulated the expression of hormone-sensitive triglyceride lipase (HSL) and adipose triglyceride lipase (ATGL) and upregulated the phosphorylation of acetyl-CoA carboxylase (ACC) related to fatty acid oxidation and lipogenesis. This dual-target compound with lipid metabolism regulatory efficacy may represent a promising type of drug lead for NAFLD therapy.

## 1. Introduction

Nonalcoholic fatty liver disease (NAFLD) is widely recognized as a universal chronic liver disease that increases the risk of severe liver diseases, including hepatic fibrosis, cirrhosis and hepatocellular carcinoma, and also is the main driver of cardiovascular diseases [1,2,3]. The lipid toxicity caused by abnormal accumulation of lipids, mainly in the form of triacylglycerols (TG), in hepatocytes characterizes the key pathogenic event in NAFLD development, which promotes inflammation and leads to hepatic injury [4,5,6,7]. And this was frequently observed in some patients with an excessively high-fat diet and obesity, or in the case of insulin resistance caused by elevated levels of free fatty acid (FFA) in the liver [8,9]. Although the pathogenesis of NAFLD is not fully understood, there is a consensus that metabolic disorders and hepatic steatosis play a key role in the initiation of the disease.

Peroxisome proliferator-activated receptors (PPARs) [10] play an important role in the regulation of lipid metabolism and also function in important ways to cure hepatic fibrosis, reduce steatosis and inflammation and alleviate the symptoms of chronic liver disease [11,12,13,14]. The PPARα isoform is predominantly expressed in hepatocytes, where it facilitates the transportation and oxidation of fatty acid and limits the inflammatory response [15,16]. In addition, PPARα could induce CPT1 expression, where the latter is a rate-limiting enzyme in fatty acid β-oxidation (FAO) [17], while mitochondrial FAO is a major route for lipid expenditure [18]. Here, the carnitine palmitoyltransferase (CPT) system is responsible for the transport of long-chain acyl-CoA esters into the mitochondria matrix [19] and CPT1 could be inhibited by malonyl-CoA, which serves as the key regulatory mechanism to maintain a steady state of fatty acid metabolism [20]. Therefore, the search for novel compounds that can activate PPARα/CPT1 and promote FAO to attenuate lipid accumulation and metabolic disorders is of substantial interest.

Although great efforts have been deployed to develop potent and selective agonists for pharmacological use in metabolic diseases [21], only two chemical families, thiazolidinediones and fibrates, are well described as lipid regulators [22,23,24]. Unfortunately, many of the PPAR agonists lead to toxic side effects.

**Figure 1:**
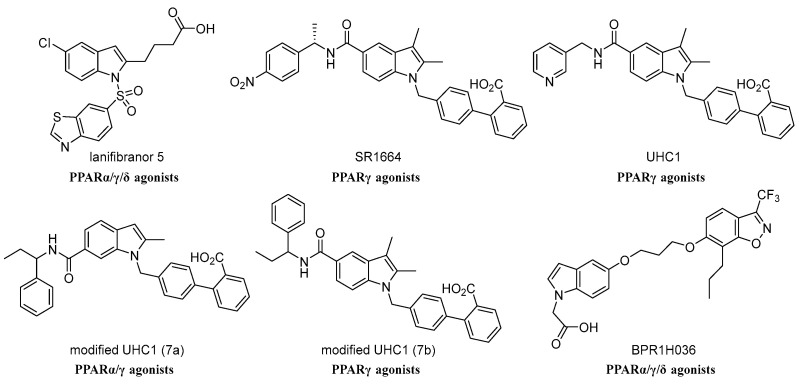
Some representative leading compounds of indole-based PPAR agonists.

Over the past few decade, scientists have amassed considerable evidence for indole-containing skeletons as a new promising class of PPAR regulator. Some indole-based derivatives were reported as agonist ligands of PPAR and are anti-diabetic in vivo [25,26]. For example, lanifibranor 5 [27], SR1664 [28], UHC1 [28], modified UHC1 (7a) [29], modified UHC1 (7b) [29], and BPR1H036 [30] (Figure 1). However, in the current study of the clinical relevance to nonalcoholic steatohepatitis (NASH), the progressive form of NAFLD, most research has focused on the efficacy of lanifibranor, a next-generation high-potential indole sulfonamide derivative, an anti-NASH drug, currently in clinical trial phase III [27], designed to target and ensure the well-balanced activation of all three subtypes of PPAR. Considering the structural characteristics of these active compounds with substitutions at N1 and C2 (Figure 1), we have designed and synthesized a new set of PPARα regulators (compounds **4**, **5**, **8**, and **9**). In this study, we have performed virtual screening by means of molecular docking studies to identify the potential active molecules with detailed structural dynamic information and an activating mechanism. We report herein the synthesis of a novel analog of indole-based derivative **9**, possessing highly potent PPARα binding affinity. This compound provides a promising approach to treat NAFLD.

## 2. Results

### 2.1. Chemistry

**Scheme 1:**
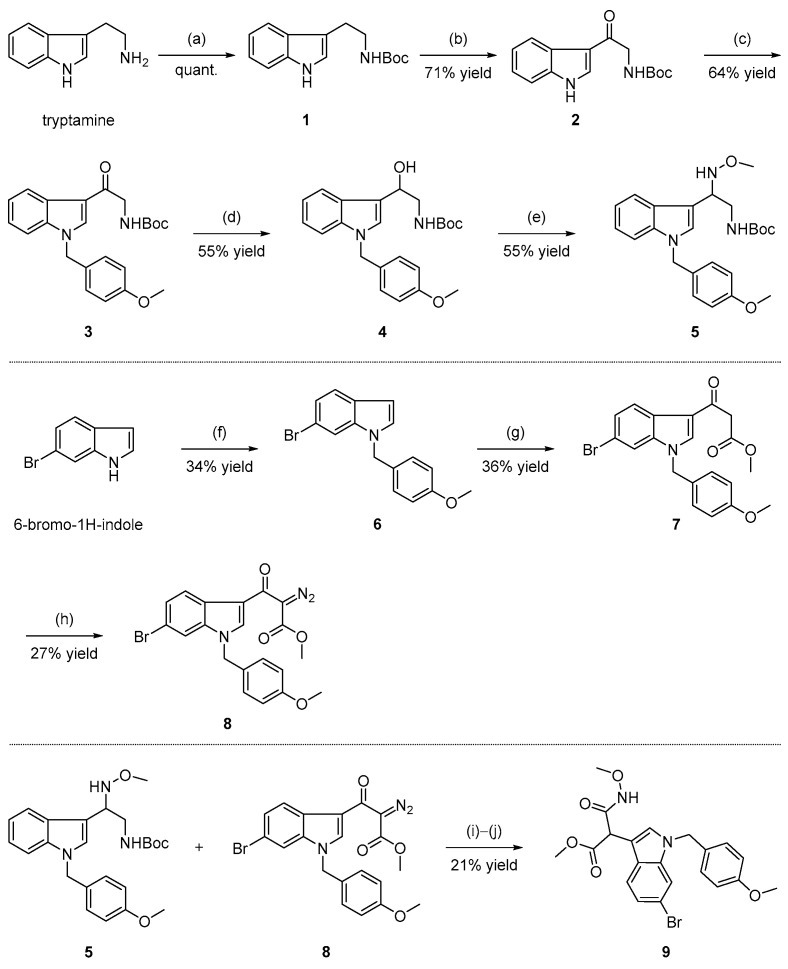
Synthetic route of compound **9**. Reagents and conditions: (**a**) (Boc)_2_O, NEt_3_, THF, rt, quant.; (**b**) DDQ, THF, H_2_O, rt, overnight, 71% yield; (**c**) PMBBr, NaH, THF, 0 °C to rt, 64% yield; (**d**) DIBAL-H, −78 °C, 55% yield; (**e**) MeONH_2_•HCl, NEt_3_, toluene, 110 °C, 55% yield; (**f**) NaH, PMBBr, THF, 0 °C to rt, 34% yield; (**g**) methyl 3-chloro-3-oxopropanoate, AlCl_3_, CH_2_Cl_2_, 0 °C to rt, 36% yield; (**h**) *p*-ABSA, NEt_3_, CH_3_CN, rt, 27% yield; (**i**) PhCF_3_, 110 °C, 37% yield; and (**j**) TMSOTf, 2,6-lutidine, CH_2_Cl_2_, 0 °C to rt, 57% yield.

**Figure 2:**
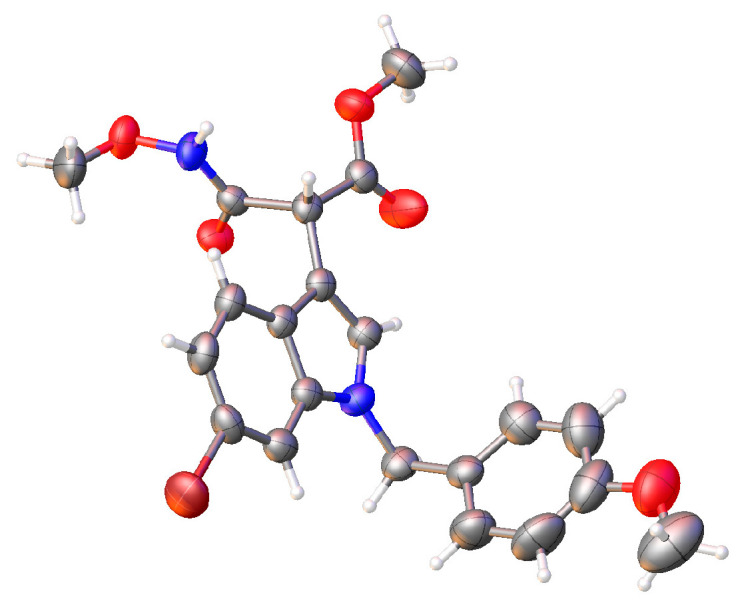
X-ray structure of compound **9**.

The general procedures for the synthesis of indole ethylamine derivatives are described in Scheme 1. We started our synthetic route with commercially available tryptamine as the starting material. Sequential transformations involving chemoselective *N*-Boc protection, benzylic oxidation, N1 protection as the *N*-PMB and the reduction of the ketone yielded the desired alcohol **4** [31,32,33]. Hydroxylamine **5** was obtained with an acceptable yield of 55% via formal nucleophilic substitution reactions of **4** and MeONH_2_•HCl in the presence of a base [34]. On the other hand, starting from 6-Br-1H-indole, the *β*-keto ester **7** was synthesized via N1 protection followed by C3 Friedel–Crafts acylation [35,36]. Regitz diazo transfer of **7** was realized using 4-acetamidobenzenesulfonyl azide (*p*-ABSA) as the azide reagent under mild conditions, generating *α*-diazo carbonyl compound **8** [37]. Coupling of **5** and **8** was achieved when the ketene derived from the Wolff rearrangement of **8** was trapped by the secondary amine of **5** to furnish *α*-amine *β*-keto ester (not shown) [38], which was exposed to TMSOTf and 2,6-lutidine to provide **9**. *N*-Boc deprotection and the following formal fragmentation might account for the unprecedented formation of the desired product **9**. The relative stereochemistry of compounds **4**, **5**, **7**, **8**, and **9** was confirmed via ^1^H NMR, ^13^C NMR, and high-resolution mass spectrometry (HRMS). Moreover, as shown in Figure 2, the structure of our target molecule **9** was unambiguously confirmed further via X-ray crystallographic analysis (Supporting Information).

### 2.2. Biological Evaluations

#### 2.2.1. PPARα Agonist Activity

**Figure 3:**
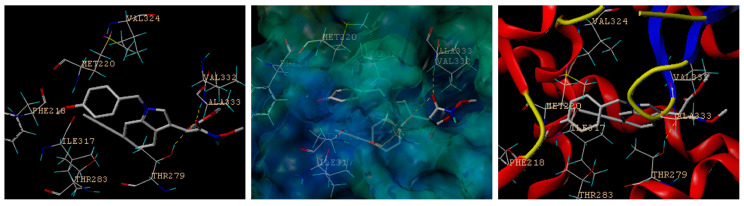
Novel indole-derived structure **9** has comparable high binding affinity to PPARα. In silico docking of novel indole ethylamine derivative **9** with the homology model of human PPARα. The compound and important amino acids in the binding pockets are shown as sticks with the following colors according to atom type: hydrogen atoms in cyan, carbon atoms in white, nitrogen atoms in blue, and oxygen atoms in red. Hydrogen bonds are represented by yellow dotted lines. A surface representation and a ribbon diagram of the PPARα structure are shown.

A new set of indole-based PPARα agonists (**4**, **5**, **8**, **9**) characterized by N1 and C2 substitutions were prepared. In order to identify the potential active structure via virtual screening, molecular docking studies were performed against PPARα in comparison with lanifibranor 5 and fenofibrate (positive control), which indicated potential for the treatment of severe hypertriglyceridemia and mixed dyslipidemia in patients who have not responded to nonpharmacological therapies. Notably, the lipid-modifying effects of fenofibrate are mediated by the activation of PPARα [39]. Consensus scoring and visual inspection of the docked complexes were used to select the optimal binding pose of the docked compounds. The highest-scoring docked position of the compounds within the drug-binding pocket of human PPARα (PDB: 6kaz.pdb) [40] is shown in Figure S1 in the Supporting Information. The docking scores of the novel indole ethylamine derivatives (**4**, **5**, **8**, **9**), fenofibrate and lanifibranor 5 binding to the drug-binding pocket of PPARα were 6.3653, 3.0505, 8.4555, 10.9630, 8.4709, and 3.4233, respectively (Figure S1), which indicates that **9** performs best in the virtual screening and has a better binding affinity to PPARα than lanifibranor 5 [27] and fenofibrate. The preferred docking orientation for compound **9** is shown in Figure 3. The docking analysis revealed the existence of hydrogen bond interactions between **9** and residues Val332 and Thr279 in the drug-binding pocket of PPARα. Moreover, it is possible that **9** is stabilized further in the drug-binding pocket of PPARα by other residues, including Val324, Ala333, Thr283, Met220, Ile317 and Phe218.

**Figure 4:**
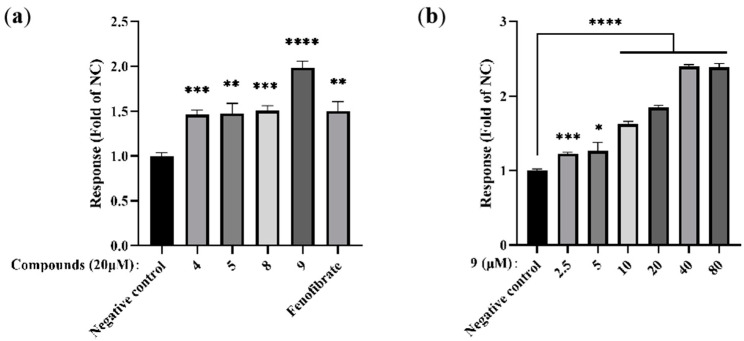
Effects of novel indole ethylamine derivatives (**4**, **5**, **8**, **9**) on PPARα agonist activity in hepatocytes. AML12 cells were treated with different concentrations of the compounds for 24 h: (**a**) PPARα levels in AML12 cells treated with all compounds (20 μM). Fenofibrate was used as the positive control; and (**b**) PPARα levels in AML12 cells treated with varying concentrations of **9** (5, 10, and 20 μM); *n* = 3. * *p* < 0.05, ** *p* < 0.01, *** *p* < 0.001, **** *p* < 0.0001 vs. the negative control group.

In order to prove the virtual molecular docking studies, the derivatives were synthesized and assayed in vitro agonist activated PPARα by using an enzyme-linked immunosorbent assay. Consistent with the molecular docking studies, indole derivative **9** showed good agonist activity on PPARα in comparison with fenofibrate (positive control) (Figure 4). The primary SAR study revealed that the C6-Br-substituted (**9** vs. **4**, **5**) substrates with a side chain containing the 1,3-dicarbonyl-2-methine motif (**9** vs. **8**) at C3 could obviously enhance the bioactivity.

#### 2.2.2. Lipid-Modifying Activity

**Figure 5:**
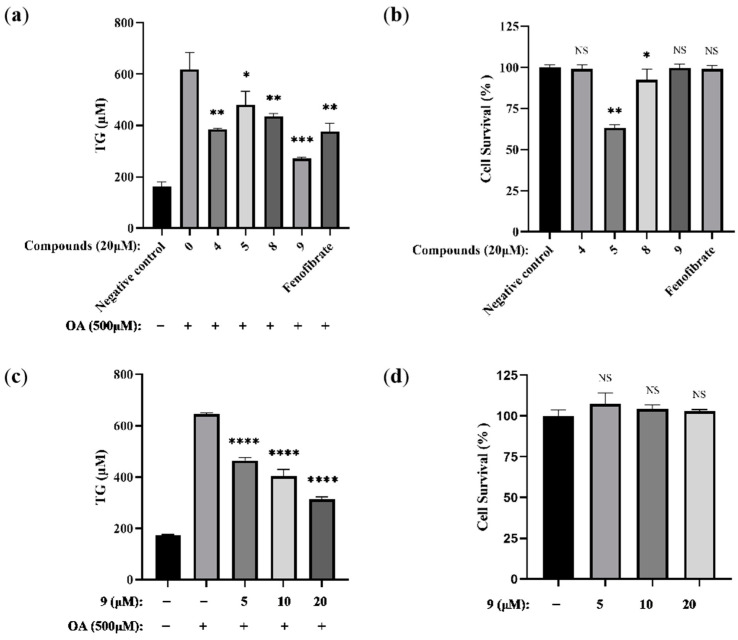
Novel indole ethylamine derivatives (**4**, **5**, **8**, **9**) prevent intracellular triglyceride (TG) accumulation in hepatocytes challenged with OA. AML12 cells were treated with different concentrations of the compounds for 2 h and then stimulated in the presence or absence of 500 μM OA for 24 h. (**a**) Hepatocyte TG levels in AML12 cells treated with all the compounds (20 μM) and/or OA. Fenofibrate was used as the positive control (*n* = 3. * *p* < 0.05, ** *p* < 0.01, *** *p* < 0.001 vs. the OA-treated control group). (**b**) Cell viability in AML12 cells treated with all the compounds (20 μM) (*n* = 4. * *p* < 0.05, ** *p* < 0.01, NS, not significant, *p* > 0.05 vs. the negative control group). (**c**) Hepatic hepatocyte TG levels in AML12 cells treated with varying concentrations of **9** (5, 10, and 20 μM) and/or OA (*n* = 3. **** *p* < 0.0001 vs. the OA-treated control group). (**d**) Cell viability in AML12 cells treated with varying concentrations of **9** (5, 10, and 20 μM) (*n* = 4. NS, not significant, *p* > 0.05 vs. the OA-treated negative control group).

The therapeutic effects of most clinical PPARα agonists on NAFLD are typically achieved by lowering the TG levels in patients. [41] To screen the indole-based PPARα agonists for their lipid-modifying effects, we investigated the effects of four new compounds (**4**, **5**, **8**, **9**) on lipid accumulation in hepatocytes via an in vitro model of oleic acid (OA)-induced triglyceride-accumulated murine hepatic AML12 cells by applying 500 µM of OA for 24 h. Adipogenesis was evaluated both by measuring the intracellular TG levels and by using fenofibrate as a positive control (Figure 5a). At the same concentration of 20 µM, all the compounds showed effects. Interestingly, compared with other compounds, compound **9** showed good activity with no observable loss of cell viability occurring (Figure 5b).

To further verify the effect of compound **9**, we next investigated whether **9** could indeed inhibit TG accumulation in hepatocytes at different concentrations. A CCK8 assay indicated that no observable loss of cell viability occurred when the concentration of **9** was equal to or below 20 μM (Figure 5d). In comparison with non-treated cells, data show **9** was demonstrated to be able to attenuate OA-promoted TG accumulation in a dose-dependent manner (Figure 5c). Since **9** has the best activity among all the tested derivatives, the underlying molecular mechanism of action study for compound **9** was elucidated in OA-induced AML12 cells.

**Figure 6:**
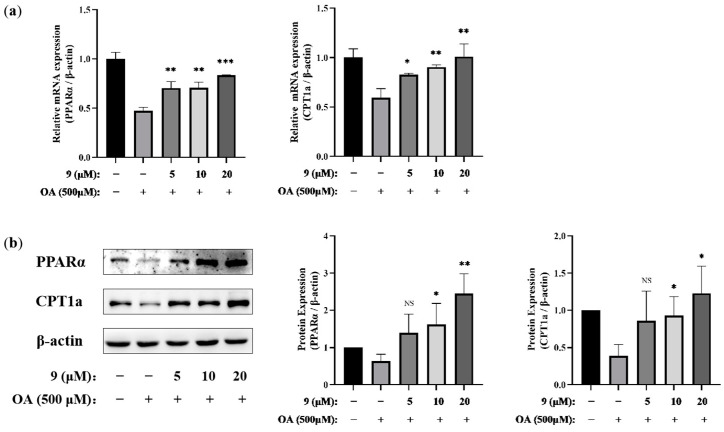
Effects of **9** on the PPARα/CPT1 pathway in OA-induced AML12 cells. AML12 cells were treated with **9** (5, 10, and 20 μM) for 2 h and then stimulated in the presence or absence of OA (500 μM) for 24 h. (**a**) The mRNA expressions of PPARα and CPT1a were analyzed via real-time RT-PCR (*n* = 3. * *p* < 0.05, ** *p* < 0.01, *** *p* < 0.001 vs. the OA-treated control group). (**b**) The protein expressions of PPARα and CPT1a were extracted and analyzed via Western blotting. β-actin was used as an internal control. The relative intensity of the indicated proteins was quantified using NIH ImageJ 1.53a software (**right** panel) (*n* = 3. * *p* < 0.05, ** *p* < 0.01, NS, not significant, *p* > 0.05 vs. the OA-treated control group).

To further verify the effect of compound **9** on the regulation of the PPARα/CPT1a signaling pathway, we investigated whether **9** could regulate PPARα/CPT1a in both the mRNA (Figure 6a) and protein levels (Figure 6b) at different concentrations. The Western blotting and PCR results indicated PPARα and CPT1a were upregulated in cells co-incubated with **9** and OA compared with OA-treated cells. PPARα and CPT1a were upregulated in cells co-incubated with 9 and OA compared with OA-treated cells in the gene and protein levels, as Western blotting (WB) and PCR indicated. Thus, after integration of the analysis, we proposed that **9** altered fatty acid metabolism, decreased lipid accumulation and relieved the corresponding lipotoxicity via the PPARα/CPT1a pathway in OA-stimulated AML12 cells.

**Figure 7:**
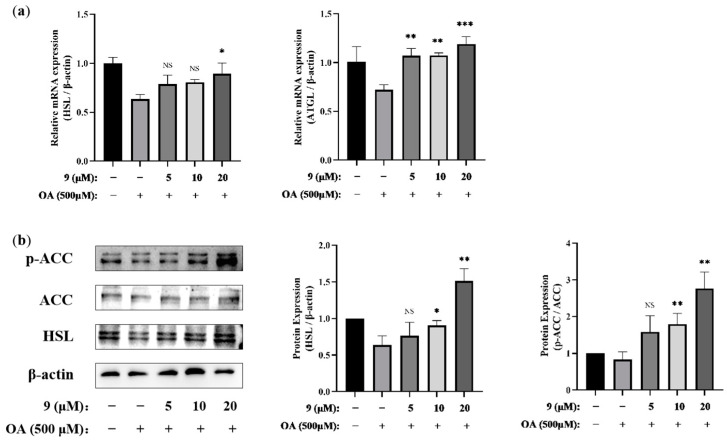
Effects of **9** on the lipid synthesis and metabolism of OA-induced AML12 cells. AML12 cells were treated with **9** (5, 10, and 20 μM) for 2 h and then stimulated in the presence or absence of OA (500 μM) for 24 h. (**a**) The mRNA expressions of HSL and ATGL were analyzed via real-time RT-PCR. *n* = 3. * *p* < 0.05, ** *p* < 0.01, *** *p* < 0.001, NS, not significant, *p* > 0.05 vs. the OA-treated control group. (**b**) The protein expressions of *p*-ACC/ACC and HSL were extracted and analyzed via Western blotting. β-actin was used as an internal control. The relative intensity of the indicated proteins was quantified using NIH ImageJ 1.53a software (**right** panel). (*n* = 3. * *p* < 0.05, ** *p* < 0.01, NS, not significant, *p* > 0.05 vs. the OA-treated control group).

The central enzyme catalyzes the first step of lipogenesis is acetyl-CoA carboxylase (ACC), which is responsible for the conversion of acetyl-CoA into malonyl-CoA, a metabolic intermediate committed to lipogenesis and an allosteric inhibitor of CPT1a. Therefore, ACC is a vital step in controlling the carbon intermediate flux from the transformation of carbohydrate and fatty acid [42]. Upregulating the expression of the phosphorylation of ACC can inhibit the synthesis of fatty acids and cholesterol. Simultaneously, by increasing the expression of fatty acid oxidation and lipid decomposition genes, like the adipose triglyceride lipase (ATGL) and hormone-sensitive triglyceride lipase (HSL) [43,44] involved in fatty acid oxidation and lipid decomposition, the lipid metabolism balance can be maintained. PCR (Figure 7a) and WB (Figure 7b) analysis showed that **9** supplementation upregulated the expression of several lipid oxidation- and lipolysis-related genes HSL, ATGL and upregulated the phosphorylation of ACC related to fatty acid oxidation and lipogenesis. 

**Figure 8:**
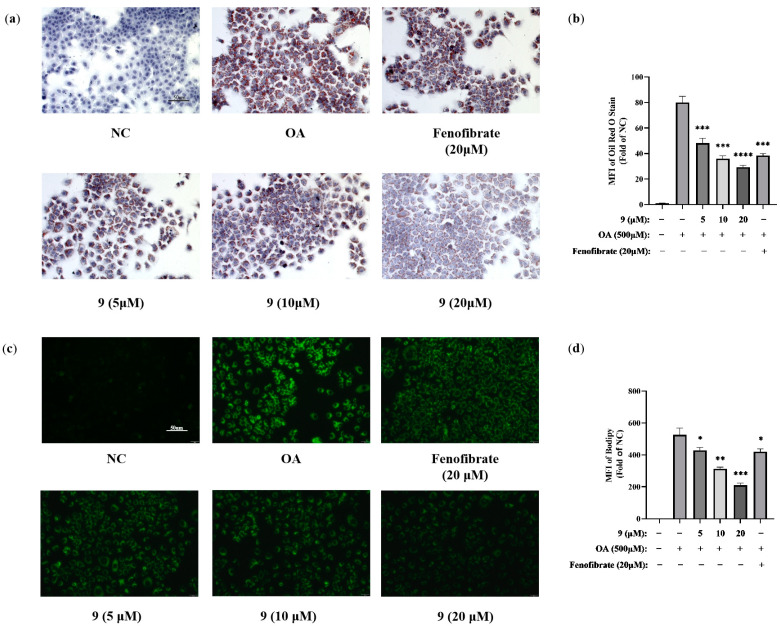
Effects of **9** on the intracellular lipid accumulation in hepatocytes challenged with OA. AML12 cells were treated with different concentrations of **9** (5, 10, and 20 μM) and 20 µM fenofibrate for 2 h and then stimulated in the presence or absence of 500 μM OA for 24 h. Fenofibrate was used as the positive control. (**a**) Oil red O staining (200×). (**b**) MFI of oil red O staining was quantified using NIH ImageJ 1.53a software. (*n* = 3. *** *p* < 0.001, **** *p* < 0.0001 vs. the OA-treated control group). (**c**) BODIPY staining (200×). (**d**) MFI of BODIPY staining was quantified using NIH ImageJ 1.53a software. (*n* = 3. * *p* < 0.05, ** *p* < 0.01, *** *p* < 0.001 vs. the OA-treated control group).

We next investigated whether **9** could indeed inhibit lipid accumulation in hepatocytes. Adipogenesis was evaluated both by measuring oil red O staining (Figure 8a,b) and by BODIPY staining (Figure 8c,d) of lipid droplets that could afford direct visual evidence of fat accumulation. As expected, **9** was demonstrated to be able to attenuate OA-promoted lipid accumulation in a dose-dependent manner.

## 3. Materials and Methods

### 3.1. Chemistry

#### 3.1.1. General Information

All the reagents were purchased from commercial sources and were used without further purification unless otherwise noted. A Perkin Elmer 650 spectrophotometer (Wal-tham, MA, USA) and a Perkin Elmer Spectrum Two FT-IR spectrometer (Waltham, MA, USA) with KBr disks were used to determine the UV and IR absorption spectra of the compounds. The NMR spectra were recorded on a 400 MHz Bruker AVANCE NEO apparatus (Basel, Switzerland) with tetramethylsilane (TMS) as an internal standard. HRESIMS data were acquired on an Agilent 6545 Q-TOF LC-MS spectrometer (Santa Clara, CA, USA) and were measured in electrospray ionization (ESI) mode, and the mass analyzer of the HRMS was the time-of-flight (TOF). A Bruker APEX DUO diffractometer (Kyoto, Japan) was used for the single crystal structure confirmation of the compounds. Flash column chromatog-raphy was performed on silica gel (200–300 mesh). After the solvent was evaporated, compound 9 (EtOAc/ Hexane1:2) was separated out as a colorless crystal at room temper-ature. The X-ray data for 9 were obtained with a Bruker APEX DUO diffractometer with Cu Kα radiation (λ = 1.54184 Å). The structures were refined and solved based on Olex2. The detailed crystallographic data of 9 are listed in the supporting information (Table S1) and deposited at the Cambridge Crystallographic Data Center with 2288643 as the deposition CCDC number.

#### 3.1.2. Synthesis of tert-butyl (2-(1H-indol-3-yl) ethyl) carbamate (**1**)

Et_3_N (6.50 mL, 46.84 mmol) was added to a yellow suspension of tryptamine (5.00 g, 31.23 mmol) in THF (20 mL). Then, 5 min later, a solution of (Boc)_2_O (8.60 mL, 37.48 mmol) in THF (5 mL) was added to the reaction mixture. The resulting mixture was stirred for 1 h. After that, the mixture was treated with the addition of H_2_O (50 mL) and AcOEt (30 mL). The organic phase was separated, and the aqueous layer was extracted with AcOEt (2 × 30 mL). The combined extracts were washed with brine (60 mL), dried over anhydrous Na_2_SO_4_, and concentrated under reduced pressure. The residue was purified via flash column chromatography on silica gel, eluting with *n*-hexane/AcOEt (7:3) to afford the desired product **1**. Yellow oil, quant. The product was characterized via the ^1^H NMR spectrum. ^1^H NMR (400 MHz, chloroform-*d*) δ = 8.54 (s, 1H), 7.62 (d, *J* = 7.9 Hz, 1H), 7.37 (d, *J* = 8.2 Hz, 1H), 7.22 (ddd, *J* = 8.2, 6.9, 1.2 Hz, 1H), 7.14 (ddd, *J* = 8.1, 7.0, 1.2 Hz, 1H), 6.99 (s, 1H), 4.75 (s, 1H), 3.49 (t, *J* = 6.7 Hz, 2H), 2.97 (t, *J* = 6.7 Hz, 2H), 1.48 (s, 9H) ppm [45].

#### 3.1.3. Synthesis of tert-butyl (2-(1H-indol-3-yl)-2-oxoethyl) carbamate (**2**)

Boc-protected tryptamine **1** (4.0 g, 15.3 mmol) was dissolved in THF/H_2_O (16:1, 34 mL). DDQ (6.91 g, 30.6 mmol, 2.0 equivalents) was added to the reaction mixture at 0 °C and the resulting mixture was stirred for 2 h at room temperature. H_2_O (50 mL) and AcOEt (30 mL) were added to the mixture. The organic phase was separated, and the aqueous layer was extracted with AcOEt (2 × 30 mL). The combined extracts were washed sequentially with saturated aqueous NaHCO_3_ (4 × 25 mL) and brine (60 mL), dried over anhydrous Na_2_SO_4_, and concentrated under reduced pressure. The residue was purified by means of flash column chromatography on silica gel, eluting with *n*-hexane/AcOEt (2:1) to afford the desired product **2**. Yellow solid, 71% yield. ^1^H NMR (400 MHz, DMSO-*d*_6_) δ = 12.01 (1H, br s, NH), 8.42 (d, *J* = 2.8, 1H), 8.22 (d, *J* = 6.1, 1H), 7.51 (d, *J* = 7.3 Hz, 1H), 7.41–7.13 (m, 2H), 7.02 (t, *J* = 7.0 Hz, 1H), 4.32 (d, *J* = 6.0 Hz, 2H), 1.43 ppm (s, 9H). ^13^C NMR (400 MHz, DMSO-*d*_6_) δ = 190.9, 156.1, 136.5, 133.4, 125.5, 122.9, 121.9, 121.3, 114.1, 112.2, 78.0, 47.0, 28.3 ppm.

#### 3.1.4. Synthesis of tert-butyl (2-(1-(4-methoxybenzyl)-1H-indol-3-yl)-2-oxoethyl) carbamate (**3**)

To a solution of compound **2** (1.0 g, 3.60 mmol) in THF (15 mL) was added sodium hydride (60% dispersion in mineral oil, 4.3 mmol) portionwise at 0 °C. Then, 30 min later, 4-methoxybenzyl bromide (4.0 mmol) was added to the reaction mixture. The resulting mixture was stirred further for 1 h at 25 °C. H_2_O (20 mL) and AcOEt (20 mL) were added to the mixture. The organic phase was separated, and the aqueous layer was extracted with AcOEt (2 × 20 mL). The combined extracts were washed with brine (60 mL), dried over anhydrous Na_2_SO_4_, and concentrated under reduced pressure. The residue was purified via flash column chromatography on silica gel, eluting with *n*-hexane/AcOEt (4:1) to afford the desired product **3**. White solid, 64% yield, mp 159–161 °C. ^1^H NMR (400 MHz, chloroform-*d*) δ = 8.33 (dd, *J* = 7.4, 1.8 Hz, 1H), 7.74 (d, *J* = 2.6 Hz, 1H), 7.38–7.34 (m, 1H), 7.29 (dt, *J* = 4.4, 2.4 Hz, 2H), 7.12 (dd, *J* = 8.8, 3.0 Hz, 2H), 6.89–6.85 (m, 2H), 5.69 (s, 1H), 5.20 (s, 2H), 4.48 (s, 2H), 3.79 (s, 3H), 1.49 ppm (s, 9H).^13^C NMR (100 MHz, chloroform-*d*) δ = 189.21, 159.79, 156.06, 137.05, 134.16, 129.07, 127.09, 126.43, 123.77, 123.10, 122.46, 114.65, 114.33, 110.46, 79.71, 55.42, 50.50, 47.64, 28.50 ppm. HRESIMS *m*/*z* = 395.1935 [M + H]^+^, calcd for 395.1965; IR (KBr) *ν*_max_ = 1701, 1651, 1517, 1465, 1389, 1251, 1168, 746, 422 cm^−1^; UV (MeOH) λ_max_ = 195, 240, 300 nm.

#### 3.1.5. Synthesis of tert-butyl (2-hydroxy-2-(1-(4-methoxybenzyl)-1H-indol-3-yl)ethyl)carbamate (**4**)

Diisobutylaluminum hydride (1.0 M solution in hexane, 2.6 mL, 2.6 mmol) was added to a solution of compound **3** (500 mg, 1.3 mmol) in THF (10 mL) at −78 °C under argon. The resulting mixture was stirred at −78 °C for 1 h. Saturated aqueous sodium potassium tartrate (5 mL) was added and the reaction mixture was stirred vigorously for 30 min. The mixture was treated with the addition of H_2_O (20 mL) and AcOEt (20 mL). The organic phase was separated, and the aqueous layer was extracted with AcOEt (2 × 20 mL). The combined extracts were washed with brine (40 mL), dried over anhydrous Na_2_SO_4_, and concentrated under reduced pressure. The residue was purified via flash column chromatography on silica gel, eluting with *n*-hexane/AcOEt (4:1) to afford the desired product **4**. White solid, 55% yield, mp 125–126 °C. ^1^H NMR (400 MHz, chloroform-*d*) δ = 7.61 (d, *J* = 7.9 Hz, 1H), 7.17 (t, *J* = 7.7 Hz, 1H), 7.08 (t, *J* = 7.7 Hz, 1H), 7.02 (d, *J* = 7.9 Hz, 1H), 6.98 (s, 1H), 6.96 (d, *J* = 8.6 Hz, 2H), 6.71 (d, *J* = 8.6 Hz, 2H), 5.08 (s, 1H, OH), 5.05 (s, 2H), 5.00 (dd, *J* = 8.7, 3.6 Hz, 1H), 3.65 (s, 3H), 3.56–3.44 (m, 1H), 3.46–3.26 (m, 1H), 1.35 ppm (s, 9H). ^13^C NMR (100 MHz, chloroform-*d*) δ = 159.06, 155.80, 136.74, 129.08, 128.30, 128.01, 125.49, 121.98, 119.46, 114.13, 114.09, 113.99, 109.88, 79.47, 68.13, 55.18, 49.48, 28.32 ppm. HRESIMS *m*/*z* 397.2160 [M + H]^+^, calcd for 397.2122; IR (KBr) *ν*_max_ = 3346, 2977, 2933, 1699, 1640, 1514, 1390, 1249, 1175, 1033, 823, 746 cm^−1^; UV (MeOH) λ_max_ = 195, 210, 250, 310 nm.

#### 3.1.6. Synthesis of tert-butyl (2-(methoxyamino)-2-(1-(4-methoxybenzyl)-1H-indol-3-yl)ethyl) carbamate (**5**)

Methoxyammonium chloride (158 mg, 1.89 mmol) and triethylamine (0.15 mL, 1.13 mmol) were sequentially added to a solution of compound **4** (150 mg, 0.37 mmol) in toluene (5 mL) at room temperature. The resulting mixture was stirred in a sealed tube overnight at 110 °C. After cooling to room temperature, the mixture was treated with the addition of H_2_O (5 mL) and AcOEt (5 mL). The organic phase was separated, and the aqueous layer was extracted with AcOEt (2 × 5 mL). The combined extracts were washed with brine (10 mL), dried over anhydrous Na_2_SO_4_, and concentrated under reduced pressure. The residue was purified via flash column chromatography on silica gel, eluting with *n*-hexane/AcOEt (2:1) to afford the desired product **5**. Yellow oil, 55% yield. ^1^H NMR (400 MHz, Chloroform-*d*) δ = 7.63 (d, *J* = 7.9 Hz, 1H), 7.20 (t, *J* = 7.8 Hz, 1H), 7.11 (t, *J* = 7.8 Hz, 1H), 7.05 (d, *J* = 7.9 Hz, 1H), 7.03 (s, 1H), 6.99 (d, *J* = 8.5 Hz, 2H), 6.74 (d, *J* = 8.5 Hz, 2H), 5.12 (s, 2H), 4.79 (d, *J* = 7.7 Hz, 1H, NH), 4.39 (t, *J* = 6.2 Hz, 1H), 3.69 (s, 3H), 3.64 (q, *J* = 5.8 Hz, 1H), 3.54–3.50 (m, 1H), 3.50 (s, 3H), 1.33 ppm (s, 9H).^13^C NMR (100 MHz, chloroform-*d*) δ = 159.01, 155.93, 136.42, 129.11, 128.19 (2C), 126.29, 121.96, 121.70, 119.42, 119.18, 114.03 (2C), 112.05, 109.85, 78.93, 62.41, 57.46, 55.07, 49.42, 43.14, 28.30 ppm. HRESIMS *m*/*z* = 426.2312 [M + H]^+^, calcd for 426.2387; IR (KBr) *ν*_max_ = 2976, 2934, 1710, 1612, 1513, 1466, 1392, 1366, 1248, 1173, 1034, 821, 741 cm^−1^; UV (MeOH) λ_max_ = 200, 230, 290 nm.

#### 3.1.7. Synthesis of 6-bromo-1-(4-methoxybenzyl)-1H-indole (**6**)

Sodium hydride (60%, dispersion in mineral oil, 6.1 mmol) was added portionwise to a mixture of 6-bromo-1H-indole (1.0 g, 1.89 mmol) in THF (10 mL). The resulting mixture was stirred for 1 h at room temperature. Then, 4-methoxybenzyl bromide (0.81 mL, 5.6 mmol) was added to the reaction mixture at 0 °C and the mixture was stirred at room temperature overnight. The reaction mixture was quenched via the dropwise addition of H_2_O (15 mL), and AcOEt (15 mL) was added to the mixture. The organic phase was separated, and the aqueous layer was extracted with AcOEt (2 × 15 mL). The combined extracts were washed with brine (30 mL), dried over anhydrous Na_2_SO_4_, and concentrated under reduced pressure. The residue was purified by means of flash column chromatography on silica gel, eluting with *n*-hexane/AcOEt (8:1) to afford the desired product **6**. Orange oil, 34% yield. ^1^H NMR (400 MHz, chloroform-*d*) δ = 7.38 (d, *J* = 8.4 Hz, 1H), 7.35 (dd, *J* = 1.7, 0.8 Hz, 1H), 7.10 (dd, *J* = 8.4, 1.7 Hz, 1H), 6.98 (d, *J* = 8.6 Hz, 1H), 6.95 (d, *J* = 3.2 Hz, 1H), 6.92 (d, *J* = 8.8 Hz, 2H), 6.72 (d, *J* = 8.7 Hz, 2H), 6.39 (dd, *J* = 3.2, 0.9 Hz, 1H), 5.05 (s, 2H), 3.65 (s, 3H) ppm.

#### 3.1.8. Synthesis of methyl 3-(6-bromo-1-(4-methoxybenzyl)-1H-indol-3-yl)-3-oxopropanoate (**7**)

Methyl malonyl chloride (0.23 mL, 2.2 mmol) was added dropwise to a mixture of compound **6** (580 mg, 1.8 mmol) and AlCl_3_ (490 mg, 3.6 mmol) in CH_2_Cl_2_ (10 mL) at 0 °C. The resulting mixture was stirred at room temperature for 11 h and treated with the careful addition of CH_2_Cl_2_ (10 mL) and H_2_O (15 mL). The organic phase was separated, and the aqueous layer was extracted with CH_2_Cl_2_ (2 × 15 mL). The combined extracts were washed with brine (20 mL), dried over anhydrous Na_2_SO_4_, and concentrated under reduced pressure. The residue was purified via flash column chromatography on silica gel, eluting with *n*-hexane/AcOEt (2:1) to afford the desired product **7**. Colorless oil, 34% yield. ^1^H NMR (400 MHz, chloroform-*d*) δ = 8.15 (d, *J* = 8.5 Hz, 1H), 7.64 (s, 1H), 7.39 (d, *J* = 1.7 Hz, 1H), 7.30 (dd, *J* = 8.5, 1.7 Hz, 1H), 7.02 (d, *J* = 8.6 Hz, 2H), 6.80 (d, *J* = 8.6 Hz, 2H), 5.11 (s, 2H), 3.74 (s, 2H), 3.71 (s, 3H), 3.64 ppm (s, 3H). ^13^C NMR (100 MHz, chloroform-*d*) δ = 186.33, 168.44, 159.82, 137.96, 135.97, 128.76 (2C), 126.81, 126.46, 125.47, 124.08, 117.59, 116.33, 114.68 (2C), 113.45, 55.44, 52.58, 50.56, 47.12 ppm. HRESIMS *m*/*z* = 438.0433 [M + 2 + Na]^+^, calcd for 438.0311; IR (KBr) *ν*_max_ = 2929, 1611, 1513, 1462, 1313, 1249, 1175, 1034, 890, 802, 718 cm^−1^; UV (MeOH) λ_max_ = 200, 230, 295 nm.

#### 3.1.9. Synthesis of methyl 3-(6-bromo-1-(4-methoxybenzyl)-1H-indol-3-yl)-2-diazo-3-oxopropanoate (**8**)

*p*-ABSA (130 mg, 0.54 mmol) and triethylamine (0.12 mL, 0.9 mmol) were sequentially added to a mixture of compound **7** (150 mg, 0.36 mmol) in MeCN (4 mL) at 0 °C. The resulting mixture was stirred at room temperature for 12 h. The resulting mixture was treated with the addition of AcOEt (4 mL) and H_2_O (5 mL). The organic phase was separated, and the aqueous layer was extracted with AcOEt (2 × 4 mL). The combined extracts were washed with brine (10 mL), dried over anhydrous Na_2_SO_4_, and concentrated under reduced pressure. The residue was purified via flash column chromatography on silica gel, eluting with *n*-hexane/AcOEt (2:1) to afford the desired product **8**. Yellow solid, 27% yield, mp 79–80 °C. ^1^H NMR (400 MHz, chloroform-*d*) δ = 8.33 (s, 1H), 8.23 (d, *J* = 8.5 Hz, 1H), 7.43 (d, *J* = 1.7 Hz, 1H), 7.36 (dd, *J* = 8.5, 1.7 Hz, 1H), 7.09 (d, *J* = 8.5 Hz, 2H), 6.85 (d, *J* = 8.5 Hz, 2H), 5.22 (s, 2H), 3.82 (s, 3H), 3.77 ppm (s, 3H).^13^C NMR (100 MHz, CDCl_3_) δ = 177.63, 162.38, 159.62, 137.57, 137.11, 128.46 (2C), 127.32, 126.95, 126.03, 123.98, 116.98, 114.53 (2C), 113.79, 113.35, 55.39, 52.24, 50.62 ppm. HRESIMS *m*/*z* = 442.0365 [M + H]^+^, calcd for 442.0397; IR (KBr) *ν*_max_ = 3140, 2954, 2838, 2144, 1720, 1611, 1578, 1514, 1468, 1369, 1294, 1249, 1177, 1105, 1034, 937, 866, 812, 743 cm^−1^; UV (MeOH) λ_max_ = 195, 220, 250, 280, 320 nm.

#### 3.1.10. Synthesis of methyl-2-(6-bromo-1-(4-methoxybenzyl)-1H-indol-3-yl)-3-(methoxyamino)-3-oxopropanoate (**9**)

Compound **5** (40 mg, 0.1 mmol) and compound **8** (49 mg, 0.12 mmol) were dissolved in PhCF_3_ (2 mL) and the resulting mixture was heated to 110 °C and stirred for 5 h. After cooling to room temperature, the mixture was treated with the addition of EtOAc (5 mL) and H_2_O (5 mL). The organic phase was separated, and the aqueous layer was extracted with AcOEt (2 × 5 mL). The combined extracts were washed with brine (10 mL), dried over anhydrous Na_2_SO_4_, and concentrated under reduced pressure. The residue was purified via flash column chromatography on silica gel, eluting with *n*-hexane/AcOEt (2:1) to afford the desired intermediate. To a solution of the intermediate (40 mg, 0.05 mmol) mentioned above in CH_2_Cl_2_ (2 mL) were added 2,6-lutidine (0.023 mL, 0.2 mmol) and TMSOTf (0.026 mL, 0.15 mmol) successively at 0 °C. The resulting mixture was stirred at room temperature for 3.5 h and treated with the careful addition of CH_2_Cl_2_ (3 mL) and H_2_O (3 mL). The organic phase was separated, and the aqueous layer was extracted with CH_2_Cl_2_ (2 × 3 mL). The combined extracts were washed sequentially with saturated aqueous sodium bicarbonate (3 mL) and brine (6 mL), dried over anhydrous Na_2_SO_4_, and concentrated under reduced pressure. The residue was purified via flash column chromatography on silica gel, eluting with *n*-hexane/AcOEt (1:1) to afford the desired product **9**. Colorless oil, 57% yield. ^1^H NMR (400 MHz, DMSO-*d*_6_) δ = 7.74 (d, *J* = 1.8 Hz, 1H), 7.54 (d, *J* = 8.5 Hz, 1H), 7.49 (s, 1H), 7.20 (d, *J* = 8.7 Hz, 2H), 7.16 (d, *J* = 8.5, 1.8 Hz, 1H), 6.88 (d, *J* = 8.7 Hz, 2H), 5.33 (s, 2H), 4.74 (s, 1H), 3.70 (s, 3H), 3.64 (s, 3H), 3.56 ppm (s, 3H). ^13^C NMR (100 MHz, DMSO) δ = 168.98, 164.06, 158.70, 136.52, 136.31, 129.59, 129.17, 128.77 (2C), 126.15, 121.92, 121.31, 114.35, 114.02 (2C), 112.98, 107.20, 63.19, 55.08, 52.38, 48.50, 46.76 ppm. HRESIMS *m*/*z* = 463.0687 [M + 2 + H]^+^, calcd for 463.0689; IR (KBr) *ν*_max_ = 3427, 2925, 2851, 2144, 1596, 1514, 1384, 1249, 1035, 866, 754, 695, 496 cm^−1^; UV (MeOH) λ_max_ = 240, 290 nm.

### 3.2. Biological Evaluations

#### 3.2.1. Molecular Modeling

All the docking studies were carried out using Sybyl-X 2.0 on a Windows workstation. The crystal structure of human PPARα (PDB: 6kaz.pdb) [40] was retrieved from the RCSB Protein Data Bank. The three-dimensional structures of the novel indole ethylamine derivatives (**4**, **5**, **8**, **9**), lanifibranor 5 and fenofibrate were constructed initially using the sketch function in Sybyl-X 2.0, followed by energy minimization using the MMFF94 force field and Gasteiger–Marsili charges. The geometry was optimized with a distance-dependent dielectric constant and a termination energy gradient of 0.001 kcal/mol employed via Powell’s method. The compounds were automatically docked into the ligand-binding pocket of PPARα using the Surflex docking program, which uses an empirical scoring function and a patented search engine. Before the docking process, the ligand was extracted and the water molecules were removed from the crystal structure. The protein was prepared using the biopolymer module implemented in Sybyl: polar hydrogen atoms were added and the automated docking manner was applied. Consensus scoring and visual inspection of the docked complexes were used to select the optimal binding pose of the docked compounds. All the docking studies were carried out as described previously [46].

#### 3.2.2. Cell Cultivation and Treatment

AML12 cells (ATCC, Manassas, VA, USA) were cultured in DMEM/F12 supplemented with 10% (*v*/*v*) fetal bovine serum (FBS), insulin-transferrin-selenium (ITS) 100× (C0341, Beyotime, Shanghai, China), and dexamethasone (D4902, Sigma-Aldrich, St. Louis, MO, USA) and incubated at 37 °C with 5% CO_2_.

#### 3.2.3. Enzyme-Linked Immunosorbent Assay

AML12 cells were inoculated into 6-well culture plates (about 1 × 10^5^–2 × 10^5^ cells/well) and cultured for 24 h. The test wells were treated with the addition of 10 μL of compound **4**, **5**, **8**, **9** and the positive control (fenofibrate), with a final concentration of 20 μM, and compound **9**, with a final concentration of 2.5 μM, 5 μM, 10 μM, 20 μM, 40 μM and 80 μM, respectively. Each sample had 3 duplicate wells, and 10 μL of blank matrix was added to the negative control wells for incubation for 24 h. The contents of the PPARα in cells were measured using a Mouse PPAR-α ELISA Kit (AB-2985A, Abmart, Shanghai, China) following the manufacturer’s protocols.

#### 3.2.4. Cell Viability Assay

AML12 cells were inoculated into 96-well culture plates (about 2 × 10^3^–4 × 10^3^ cells/well) and cultured for 24 h. The test wells were treated with the addition of 10 μL of compound **4**, **5**, **8**, **9** and fenofibrate, with a final concentration of 20 μM, and compound **9**, with a final concentration of 5 μM, 10 μM and 20 μM, respectively. Each sample had 4 duplicate wells, and 10 μL of blank matrix was added to the negative control wells for incubation for 24 h. The cell viability was determined using a Cell Counting Kit-8 (K1018, APExBIO, Houston, TX, USA) according to the manufacturer’s instructions.

#### 3.2.5. Triglyceride Assay

AML12 cells were inoculated into 6-well culture plates and cultured for 24 h. The test wells were treated with the addition of 10 μL of compound **4**, **5**, **8**, **9** and fenofibrate, with a final concentration of 20 μM, and compound **9**, with a final concentration of 5 μM, 10 μM, and 20 μM, respectively. Each sample had 3 duplicate wells, and 10 μL of blank matrix was added to the negative control wells and the OA-treated control wells for incubation for 2 h. Then, it was stimulated in the presence or absence of OA (500 μM) (Sigma-Aldrich, St. Louis, MO, USA) for 24 h to stimulate intracellular lipid accumulation. After 24 h of treatment, a Triglyceride Enzymatic Assay Kit (A110-1-1, Nanjing Jiancheng Bioengineering Institute, Nanjing, China) was used to examine the TG level in the lysed cells. Calibration of the cell protein concentration was performed via the BCA method.

#### 3.2.6. Oil Red O Staining

AML12 cells were inoculated into 6-well culture plates and cultured for 24 h. The test wells were treated with the additional of 10 μL of compound **9**, with a final concentration of 5 μM, 10 μM, and 20 Μm, and fenofibrate, with a final concentration of 20 Μm, respectively. Each sample had 3 duplicate wells, and 10 μL of blank matrix was added to the negative control wells and the OA-treated control wells for incubation for 2 h. Then, it was stimulated in the presence or absence of OA (500 μM) for 24 h to stimulate intracellular lipid accumulation. Oil red O staining solution (C0158S, Beyotime, Shanghai, China) was added to the treated cell samples for 10–20 min after washing, and hematoxylin staining solution was used for the nuclear staining. The results of the lipid accumulation were observed under a microscope.

#### 3.2.7. BODIPY Staining

After 24 h of treatment, Bodipy 493/503 (2 mg/mL in medium) (CM02294, Proteintech, Rosemont, IL, USA) was added to the treated AML12 cells and incubated at 37 °C for 30 min. Then, the surface-associated BODIPY was washed with PBS solution. Finally, the fluorescence was measured using a fluorescence microscope (ZEISS, Oberkochen, Germany) Ex/Em = 488/510 nm (FITC filter).

#### 3.2.8. Quantitative Real-Time PCR

PCR was performed as described previously [47]. After 24 h of treatment, the total RNA of the cells was collected using a SteadyPure RNA Extraction Kit (AG21024, Accurate Biology, Beijing, China). Then, the RNA was reverse transcribed into cDNA via the MonScriptTM RTIII All-in-One Mix with dsDNase (MR05101S, Monad, Wuhan, China). The primers were synthetized by Sangon Biotech Co., Ltd. (Shanghai, China). The primers used are shown in Table 1. Real-time PCR was performed using the iQ-SYBR Green PCR Supermix (Bio-Rad, Hercules, CA, USA) on a Step-One-Plus real-time PCR system (Thermo Fisher Scientific, Waltham, MA, USA). The relative expression of the target genes was analyzed via the 2^−ΔΔCt^ method and the target RNA levels were normalized using β-actin. The sequences of the primers are listed below.

**Table 1:** The sequences of the primers.

Species	Primer Target	Sequence 5′ to 3′
Mus	*PPARα*-F	AGAGCCCCATCTGTCCTCTC
	PPARα-R	ACTGGTAGTCTGCAAAACCAAA
Mus	*ATGL*-F	CAACGCCACTCACATCTACGG
	*ATGL*-R	GGACACCTCAATAATGTTGGCAC
Mus	*HSL*-F	GCTCATCTCCTATGACCTACGG
	*HSL*-R	TCCGTGGATGTGAACAACCAGG
Mus	*CPT1a*-F	CTATGCGCTACTCGCTGAAGG
	*CPT1a*-R	GGCTTTCGACCCGAGAAGA
Mus	*β-actin*-F	TTCGTTGCCGGTCCACACCC
	*β-actin*-R	GCTTTGCACATGCCGGAGCC

#### 3.2.9. Western Blotting

Western blotting was performed as described previously [48]. After 24 h of treatment, the total protein was obtained from the cells via RIPA buffer (AR0105, Boster Biological Technology, Wuhan, China) with a proteinase and phosphatase inhibitor (4906845001, Roche, Basel, Switzerland). The cells were homogenized in RIPA buffer containing protease inhibitors. The cell lysates were separated using 10% SDS-PAGE and transferred onto a polyvinylidene fluoride membrane. The membrane was incubated with the appropriate primary and secondary antibodies. The bound antibody was visualized via chemiluminescence. The following primary antibodies were used: ACC (sc-137104, Santa Cruz Biotechnology, Dallas, TX, USA), p-ACC (sc-271965, Santa Cruz Biotechnology, Dallas, TX, USA), HSL (sc-74489, Santa Cruz Biotechnology, Dallas, TX, USA), PPARα (sc-398394, Santa Cruz Biotechnology, Dallas, TX, USA), CPT1a (ab234111, Abcam, Cambridge, UK), and β-actin (ac026, ABclonal Technology Co., Ltd., Wuhan, China).

#### 3.2.10. Statistical Analysis

The data were presented as the mean ± SD for at least three independent experiments. Statistical significance (*** *p* < 0.001, ** *p* < 0.01, * *p* < 0.05) was assessed using Student’s *t*-test or a one-way analysis of variance (ANOVA) coupled with Dunnett’s *t*-test.

## 4. Conclusions

In summary, a sequence of indole-based PPARα regulators characterized by substitutions at N1 and C3 were rationally designed and efficiently synthesized. The following virtual molecular docking studies together with enzyme-linked immunosorbent assays revealed that PPARα could be increased by all of the chemically synthesized derivatives. Of note, the 1,3-dicarbonyl compound **9** with Br-substituted at C6 gave the optimal activity with no observable cytotoxicity. Particularly, **9** showed more activity than the positive control fenofibrate in terms of the lipid-modifying effects and had an important effect on lipid metabolism regulation by targeting PPARα/CPT1 in OA-induced AML12 cells. Lastly, PCR and WB analysis demonstrated that **9** supplementation upregulated the expression of several lipid oxidation- and lipolysis-related genes HSL, ATGL and the phosphorylation of ACC related to fatty acid oxidation and lipogenesis. These results indicate that this dual-target compound with lipid metabolism regulatory efficacy may represent a promising type of drug lead for NAFLD therapy.

## Data Availability

Data are contained within the article and Supplementary Materials.

## Supplementary Materials

The following supporting information can be downloaded at https://www.mdpi.com/article/10.3390/molecules29010012/s1, Figure S1: Novel indole-derived structures (**4**, **5**, **8**, **9**), fenofibrate and flanifibranor 5 have comparable high binding affinities to PPARα; Figures S2–S32: ^1^H, ^13^C, IR, UV and HRESIMS data for compounds **3**–**9**; Figure S33: X-ray molecular structure of **9**; Table S1: The crystallographic data of **9**.
