# Supplementary material for: Design and Synthesis of Novel Indole Ethylamine Derivatives as a Lipid Metabolism Regulator Targeting PPARα/CPT1 in AML12 Cells
Source: Molecules. 2023 Dec 19;29(1):12. doi: 10.3390/molecules29010012 (PMC10779794; doi:10.3390/molecules29010012)
Supplement: Supplementary file 1 [file molecules-29-00012-s001.zip › molecules-2742443-supplementary.pdf]

Supplementary Data

Design and Synthesis of Novel Indole Ethylamine Derivatives as a Lipid Metabolism Regulator Targeting PPARα/CPT1 in AML12 Cells

Yu-Chen Liu [1,†], Gang Wei [2,†], Zhi-Qiang Liao [2], Fang-Xin Wang [2], Chunxiao Zong [1], Jiannan Qiu [1], Yifei Le [1], Zhi-Ling Yu [3], Seo Young Yang [4], Heng-Shan Wang [2], Xiao-Bing Dou [1,*] and Cai-Yi Wang [1,*]

[1] College of Life Science, Zhejiang Chinese Medical University, Hangzhou 310053, China; 17774008010@163.com (Y.-C.L.); z1c10x1@163.com (C.Z.); qjntcm@163.com (J.Q.); lyf19970605@foxmail.com (Y.L.)

[2] State Key Laboratory for Chemistry and Molecular Engineering of Medicinal Resources/Key Laboratory for Chemistry and Molecular Engineering of Medicinal Resources (Ministry of Education of China), School of Chemistry and Pharmaceutical Sciences, Collaborative Innovation Center for Guangxi Ethnic Medicine, Guangxi Normal University, Guilin 541004, China; wei18249913020@163.com (G.W.); 15077302639@163.com (Z.-Q.L.); wangfx@mailbox.gxnu.edu.cn (F.-X.W.); whengshan@163.com (H.-S.W.)

[3] School of Chinese Medicine, Hong Kong Baptist University, Hong Kong, China; zlyu@hkbu.edu.hk

[4] Department of Biology Education, Teachers College and Institute for Phylogenomics and Evolution, Kyungpook National University, Daegu 41566, Republic of Korea; syy@knu.ac.kr

* Correspondence: cloudwater@zcmu.edu.cn (X.-B.D.); wangcaiyiamy@163.com (C.-Y.W.); Tel.: +86-0571-86613598 (X.-B.D.); +86-0571-86613598 (C.-Y.W.)

† These authors contributed equally to this work.

content

Figure S1. Novel indole-derived structures (**4**, **5**, **8**, **9**), fenofibrate and flanifibranor 5 have comparable high binding affinities to PPARα. 1
Figure S2. The HRESIMS spectrum of **3**. 2
Figure S3. The IR spectrum of **3** 3
Figure S4. The UV spectrum of **3** (MeOH) 3
Figure S5. The ^{1}H NMR spectrum (chloroform-*d*, 400MHz) of **3**. 4
Figure S6. The ^{13}C NMR spectrum (chloroform-*d*, 400MHz) of **3**. 5
Figure S7. The HRESIMS spectrum of **4**. 5
Figure S8. The IR spectrum of **4**. 5
Figure S9. The UV spectrum of **4** (MeOH). 6
Figure S10. The ^{1}H NMR spectrum (chloroform-*d*, 400MHz) of **4**. 6
Figure S11. The ^{13}C NMR spectrum (chloroform-*d*, 400MHz) of **4**. 7
Figure S12. The HRESIMS spectrum of **5** 8
Figure S13. The IR spectrum of **5**. 8
Figure S14. The UV spectrum of **5** (MeOH). 9
Figure S15. The ^{1}H NMR spectrum (chloroform-*d*, 400MHz) of **5**. 9
Figure S16. The ^{13}C NMR spectrum (chloroform-*d*, 400MHz) of **5**. 10
Figure S17. The ^{1}H NMR spectrum (chloroform-*d*, 400MHz) of **6**. 10
Figure S18. The HRESIMS spectrum of **7**. 11
Figure S19. The IR spectrum of **7**. 11
Figure S20. The UV spectrum of **7** (MeOH). 12
Figure S21. The ^{1}H NMR spectrum (chloroform-*d*, 400MHz) of **7**. 12
Figure S22. The ^{13}C NMR spectrum (chloroform-*d*, 400MHz) of **7**. 13
Figure S23. The HRESIMS spectrum of **8**. 14
Figure S24. The IR spectrum of **8**. 14
Figure S25. The UV spectrum of **8** (MeOH). 15
Figure S26. The ^{1}H NMR spectrum (chloroform-*d*, 400MHz) of **8** 16
Figure S27. The ^{13}C NMR spectrum (chloroform-*d*, 400MHz) of **8** 16
Figure S28. The HRESIMS spectrum of **9** 17
Figure S29. The IR spectrum of **9**. 17
Figure S30. The UV spectrum of **9** (MeOH) 18
Figure S31. The ^{1}H NMR spectrum (DMSO-d_6, 400MHz) of **9** 19
Figure S32. The ^{13}C NMR spectrum (DMSO-d_6, 400MHz) of **9** 19
Figure S33. X-ray molecular structure of **9** 20
Table S1. The crystallographic data of **9** 20

4

5

8

9

Flanifibranor 5

Fenofibrate

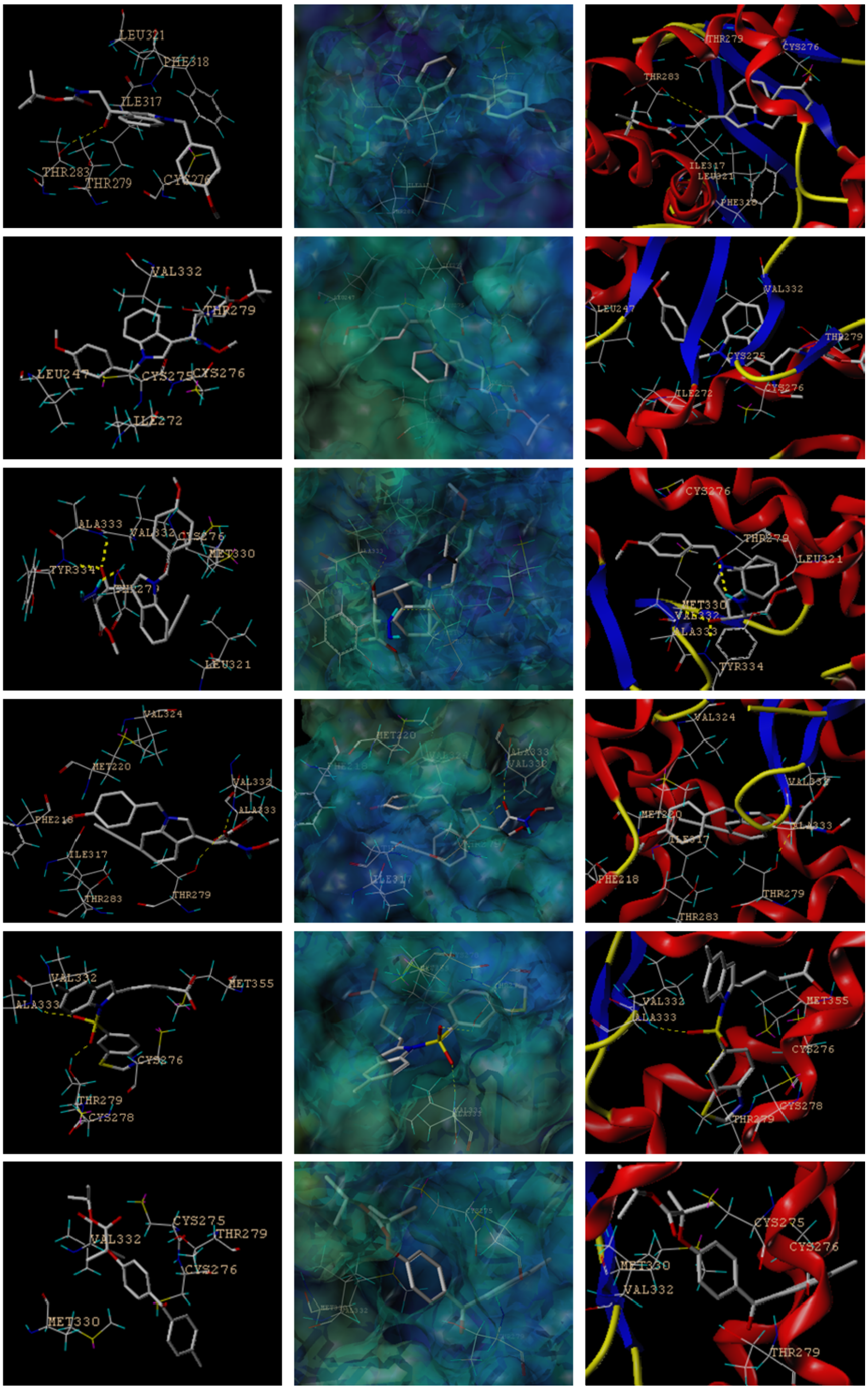

Figure S1. Novel indole-derived structures (**4**, **5**, **8**, **9**), fenofibrate and flanifibranor 5 have comparable high binding affinities to PPARα.

tert-butyl (2-(1-(4-methoxybenzyl)-1H-indol-3-yl)-2-oxoethyl)carbamate of (3)

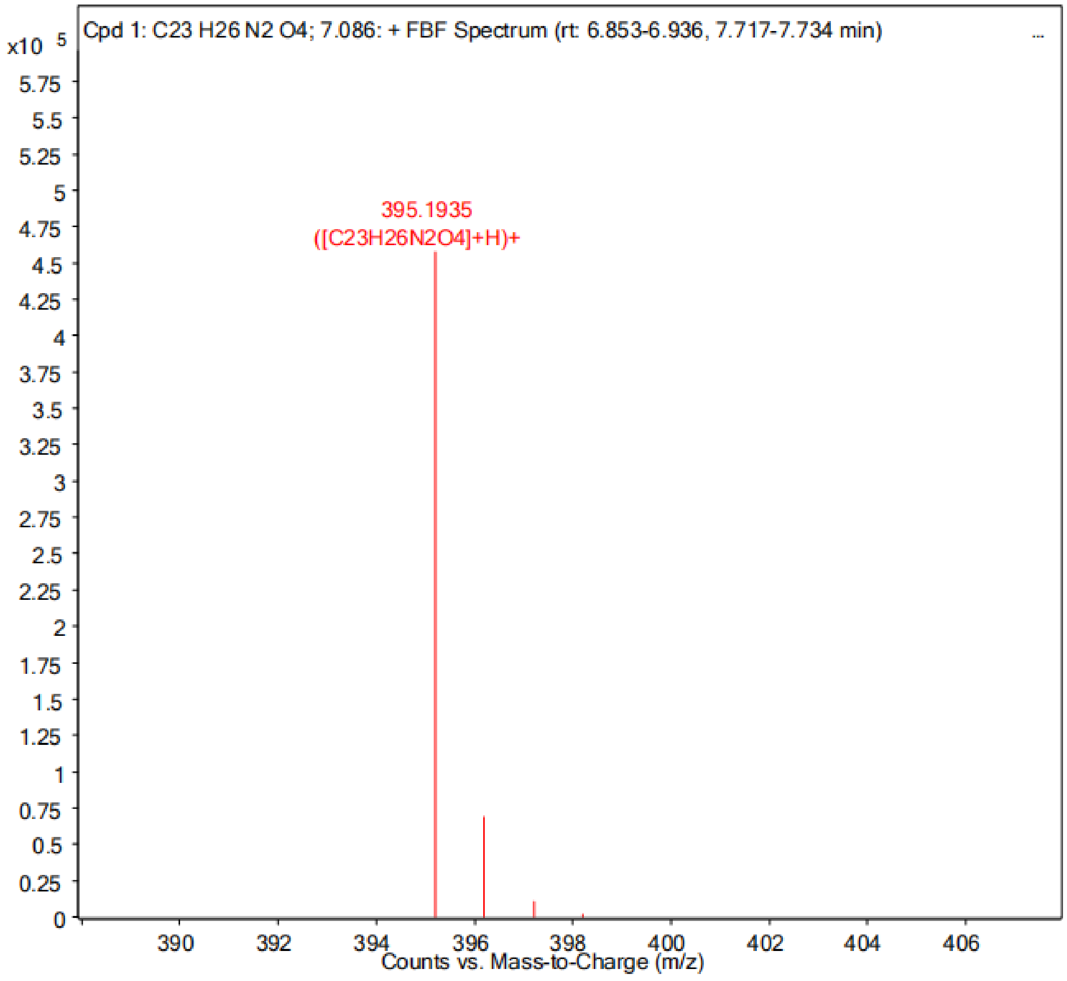

Figure S2. The HRESIMS spectrum of **3**

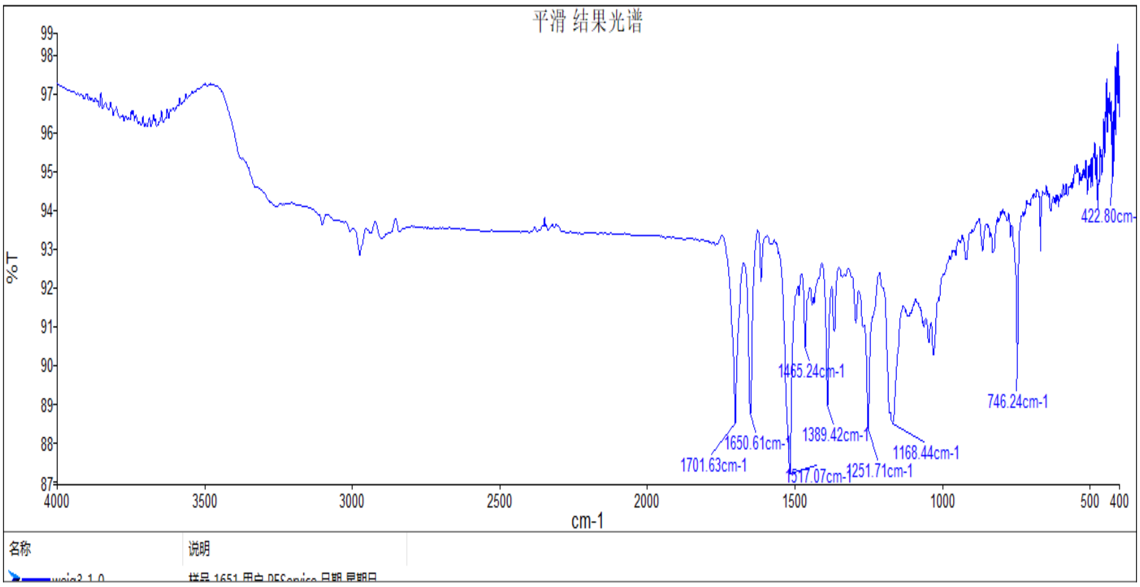

Figure S3. IR (KBr) Spectrum of compound **3**

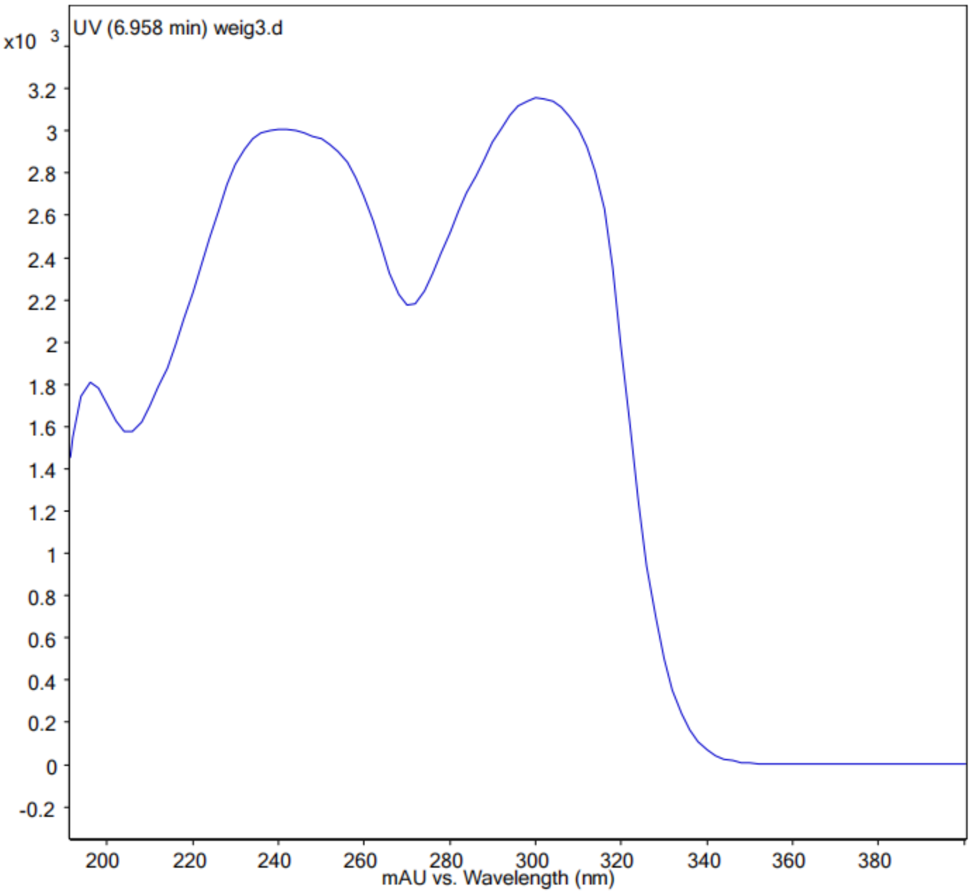

Figure S4. The UV spectrum of **3** (MeOH)

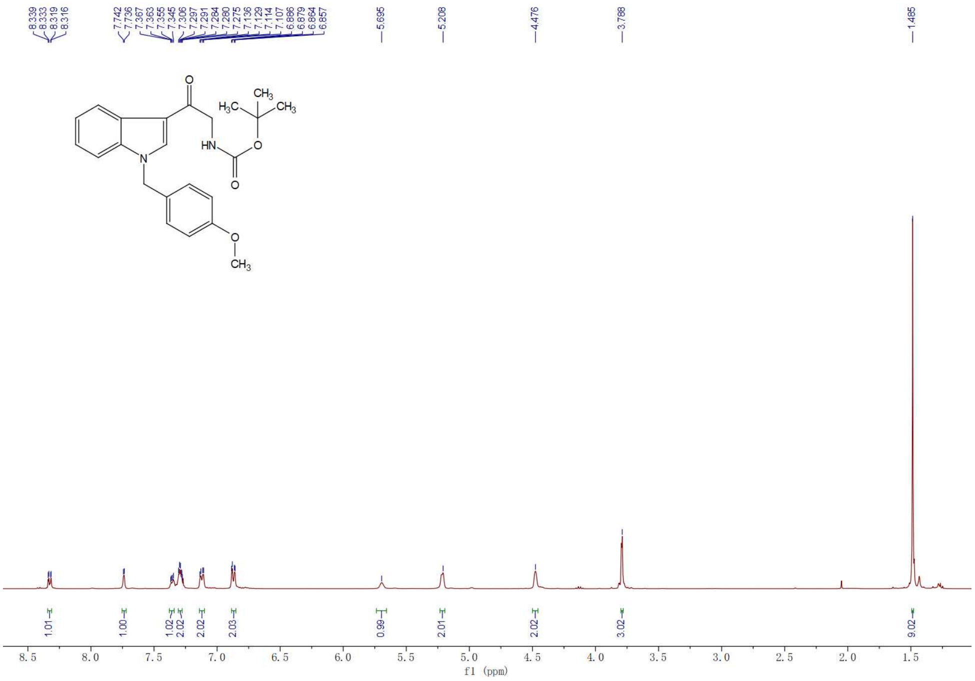

Figure S5. The ^{1}H NMR spectrum (400 MHz, chloroform-*d*) of **3**

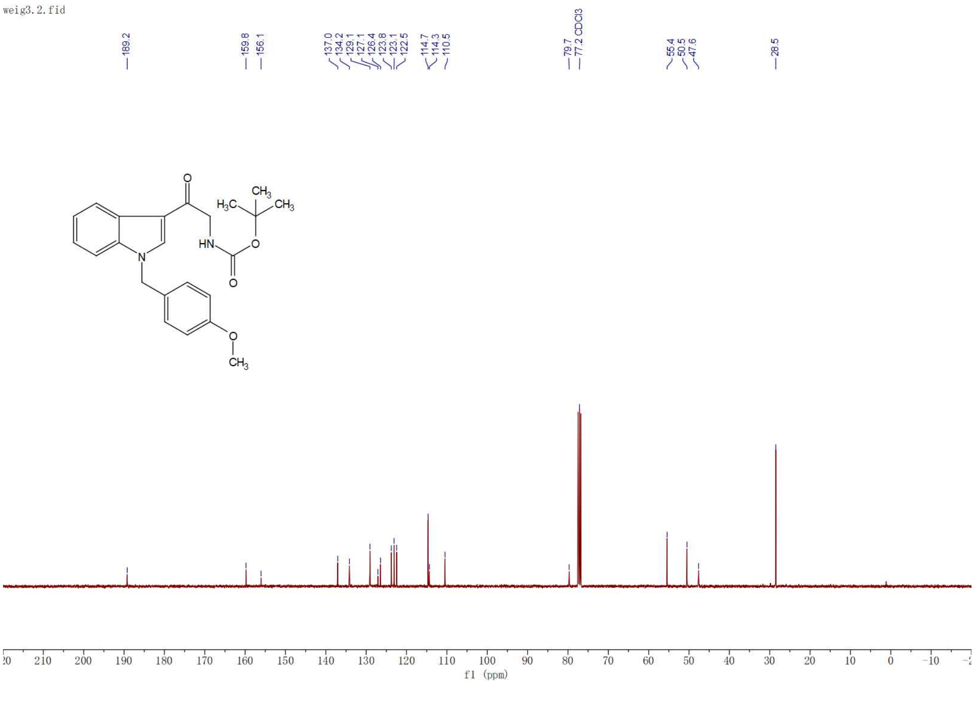

Figure S6. The ^{13}C NMR spectrum (400 MHz, chloroform-*d*) of **3**

tert-butyl (2-hydroxy-2-(1-(4-methoxybenzyl)-1H-indol-3-yl)ethyl)carbamate (4)

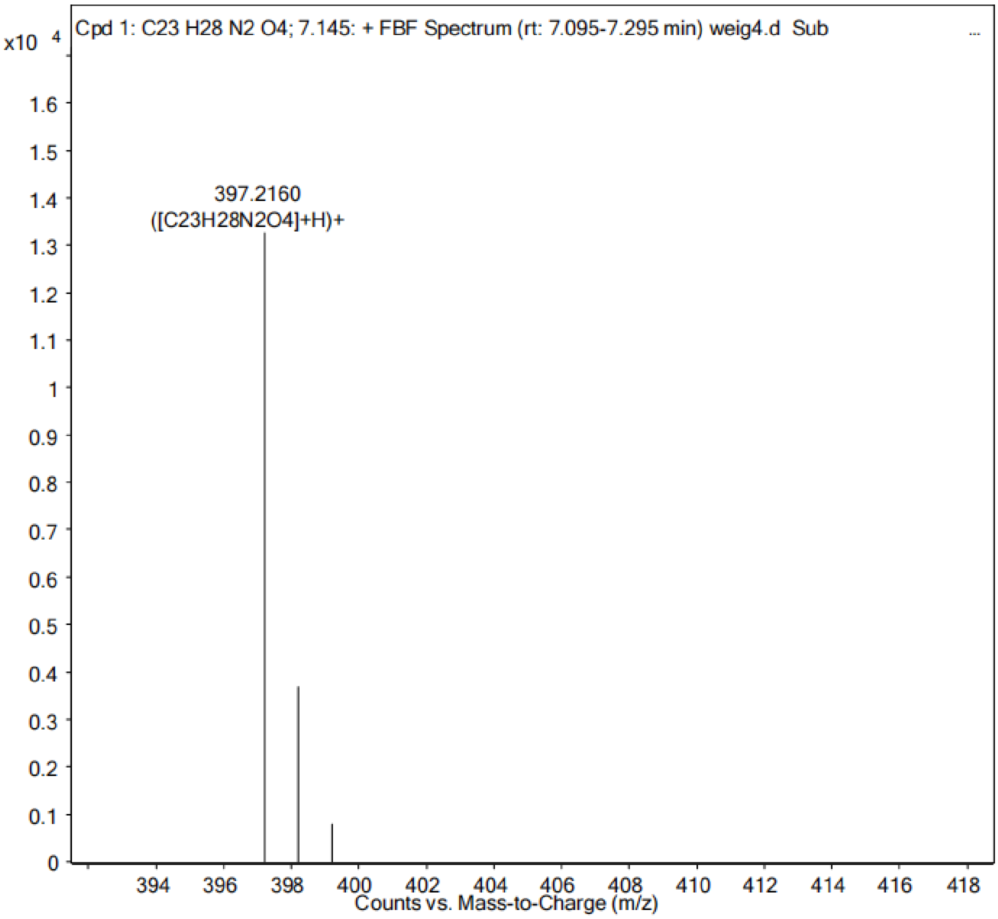

Figure S7. The HRESIMS spectrum of **4**

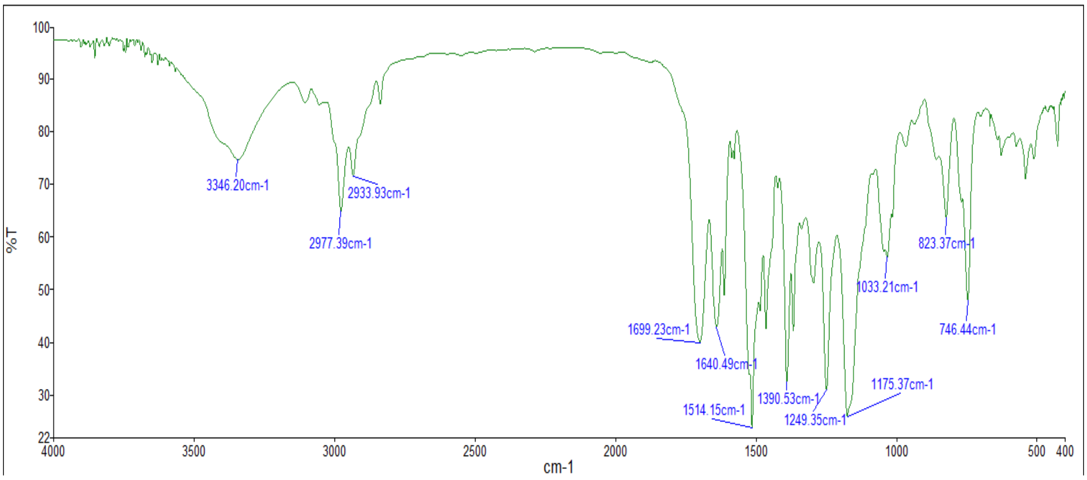

Figure S8. IR (KBr) spectrum of compound **4**

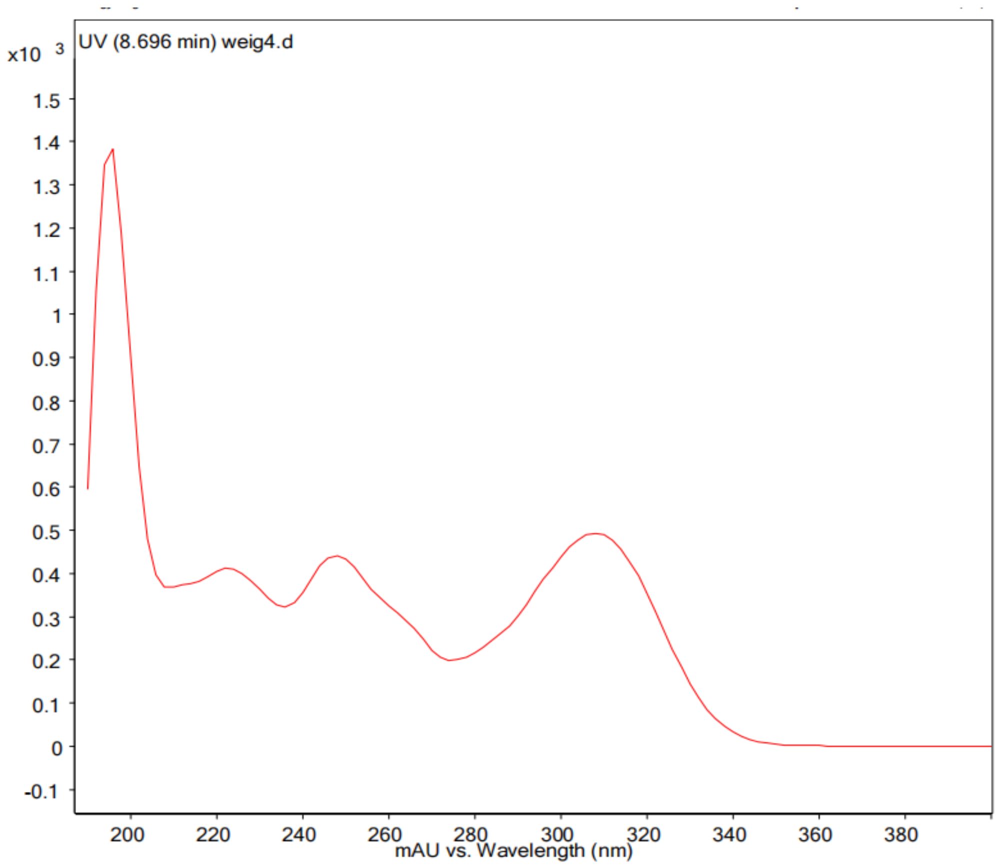

Figure S9. The UV spectrum of **4** (MeOH)

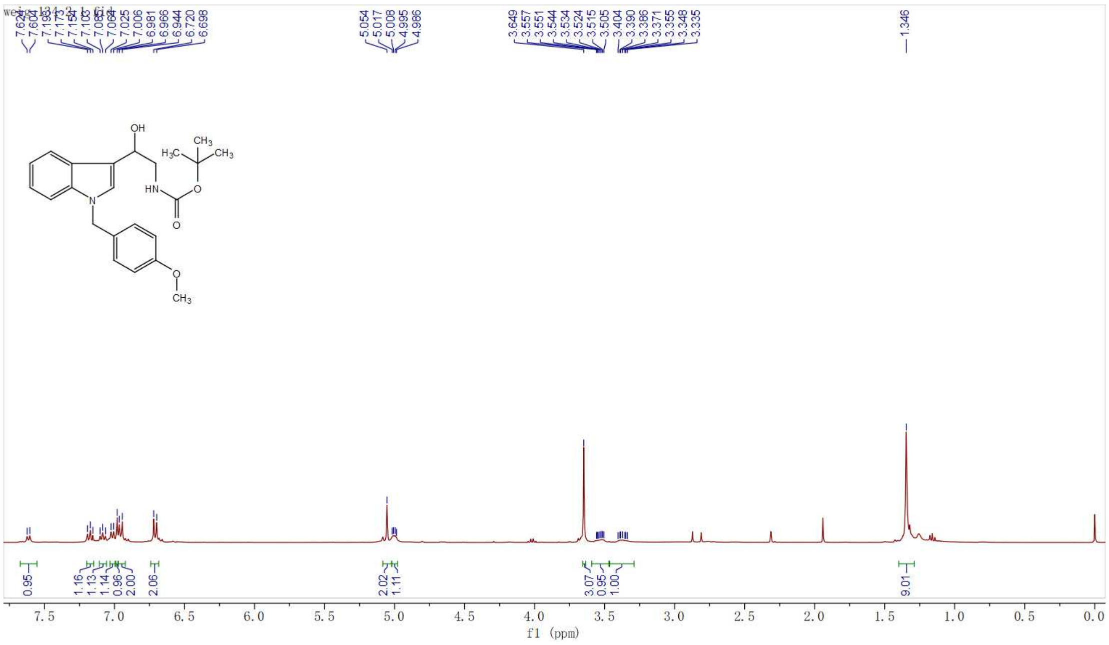

Figure S10. The ^{1}H NMR spectrum (400 MHz, chloroform-*d*) of **4**

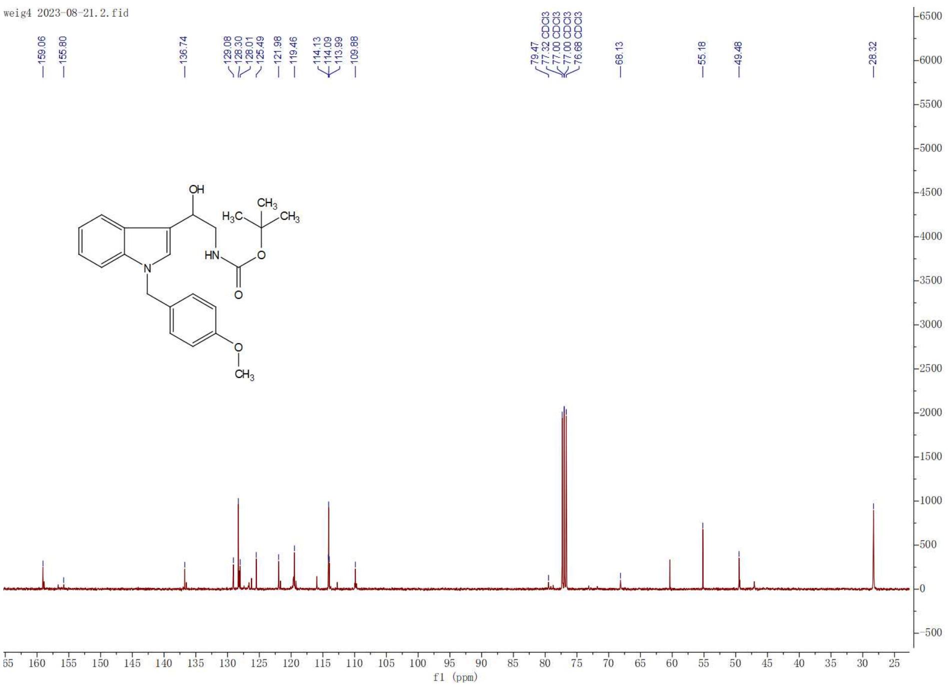

Figure S11. The ^{13}C NMR spectrum (400 MHz, chloroform-*d*) of **4**

tert-butyl(2-(methoxyamino)-2-(1-(4-methoxybenzyl)-1H-indol-3-yl)ethyl)carbamate (5)

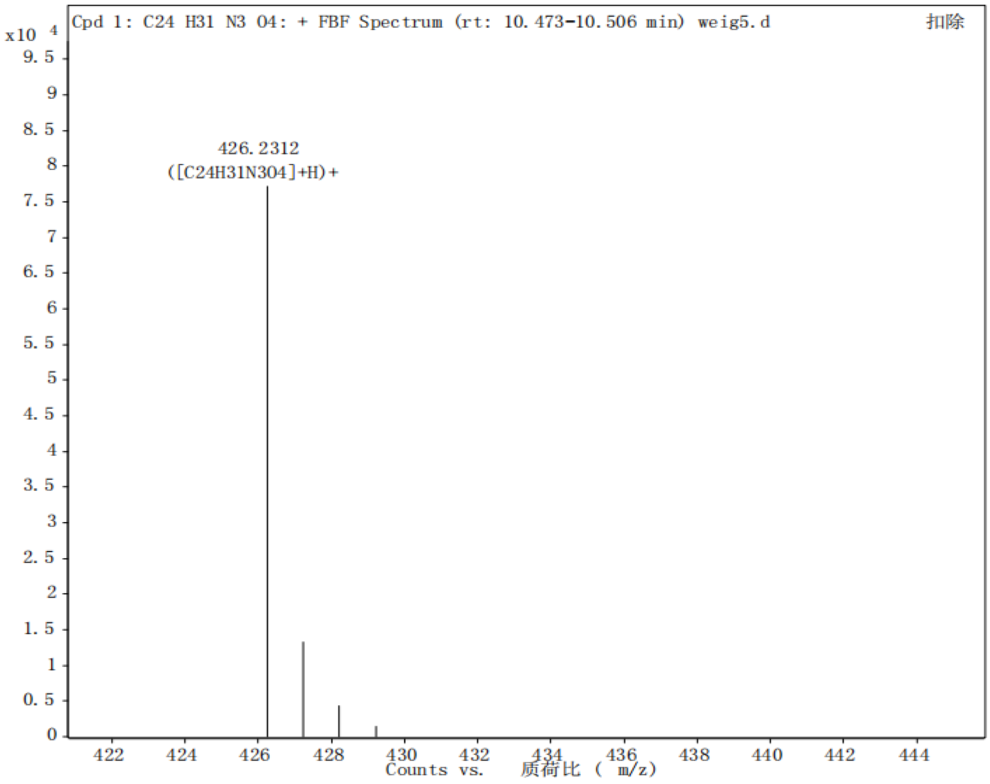

Figure S12. The HRESIMS spectrum of **5**

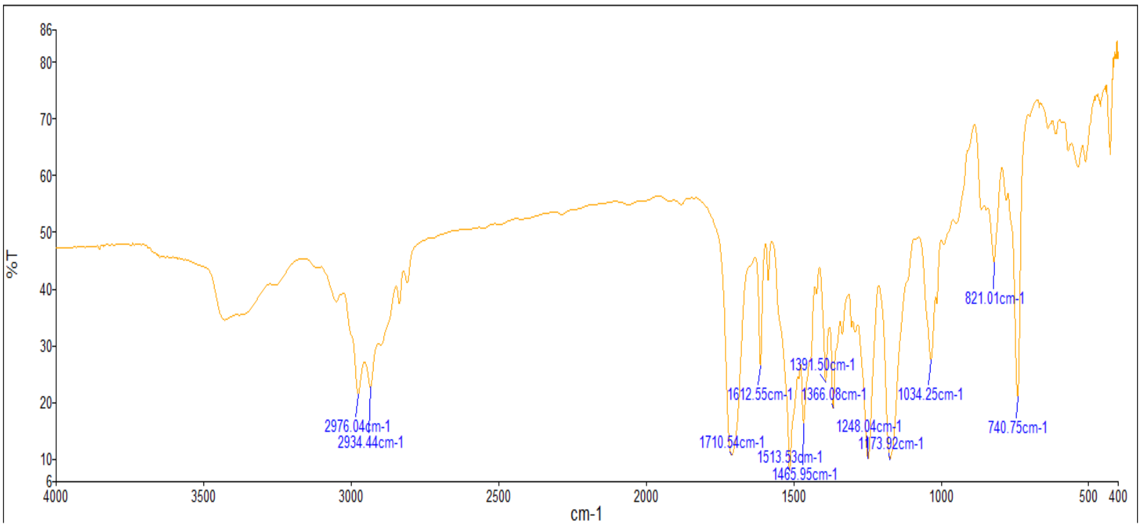

Figure S13. IR (KBr) spectrum of compound **5**

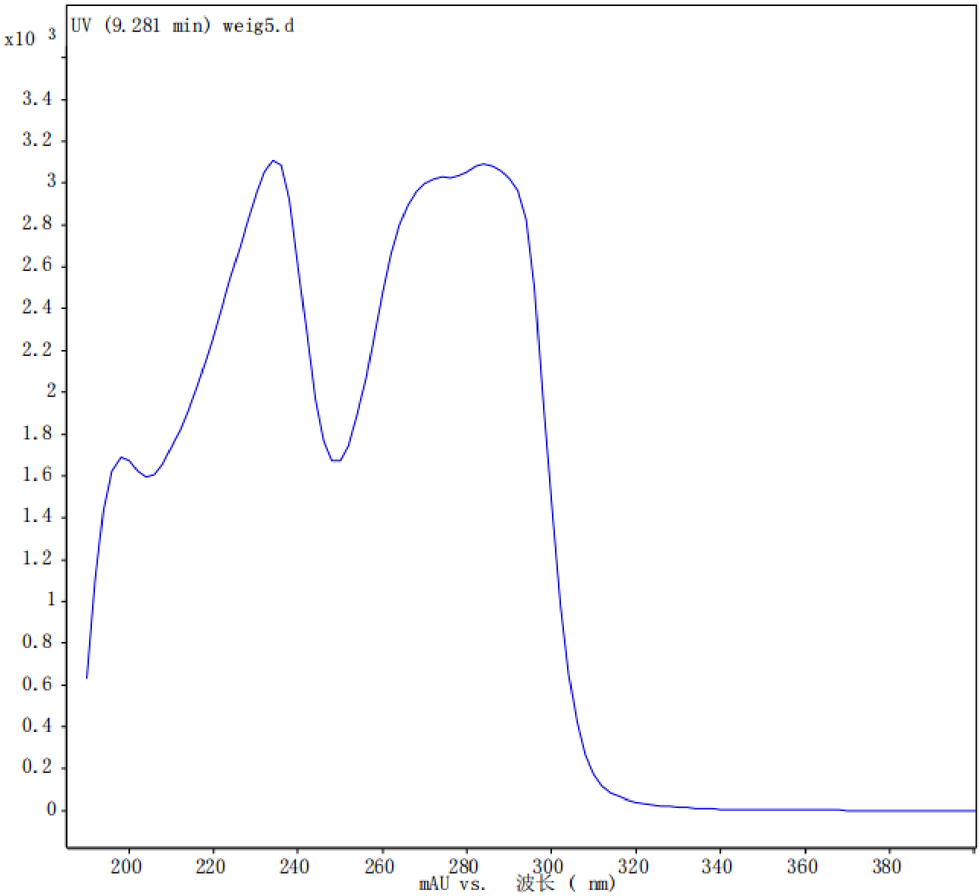

Figure S14. The UV spectrum of **5** (MeOH)

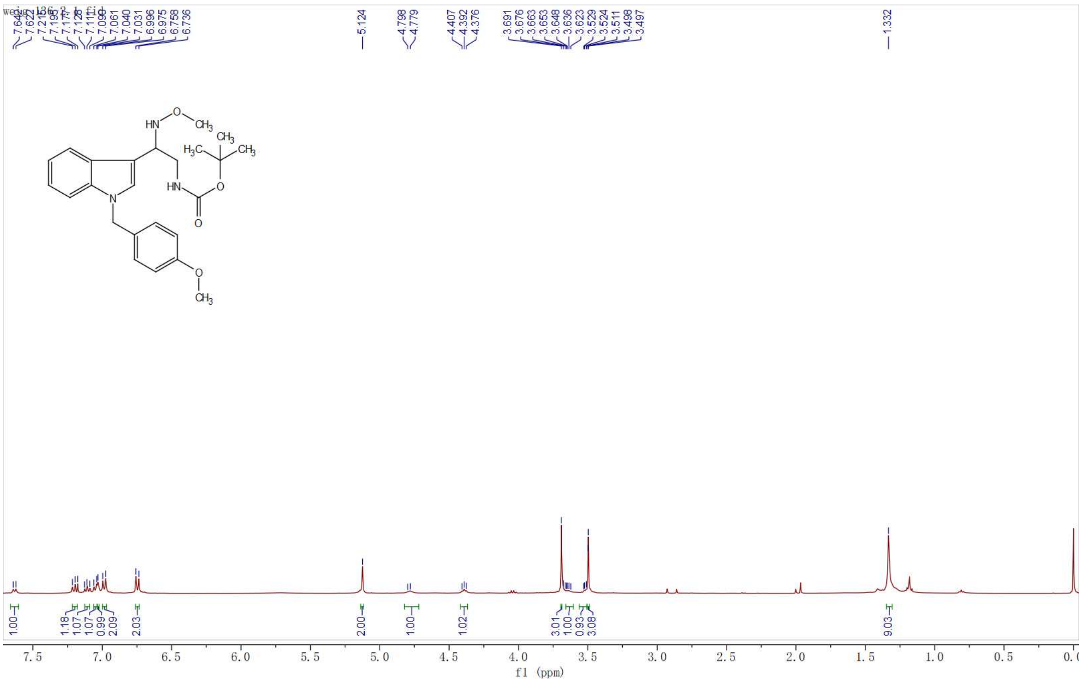

Figure S15. The ^{1}H NMR spectrum (400 MHz, chloroform-*d*) of **5**

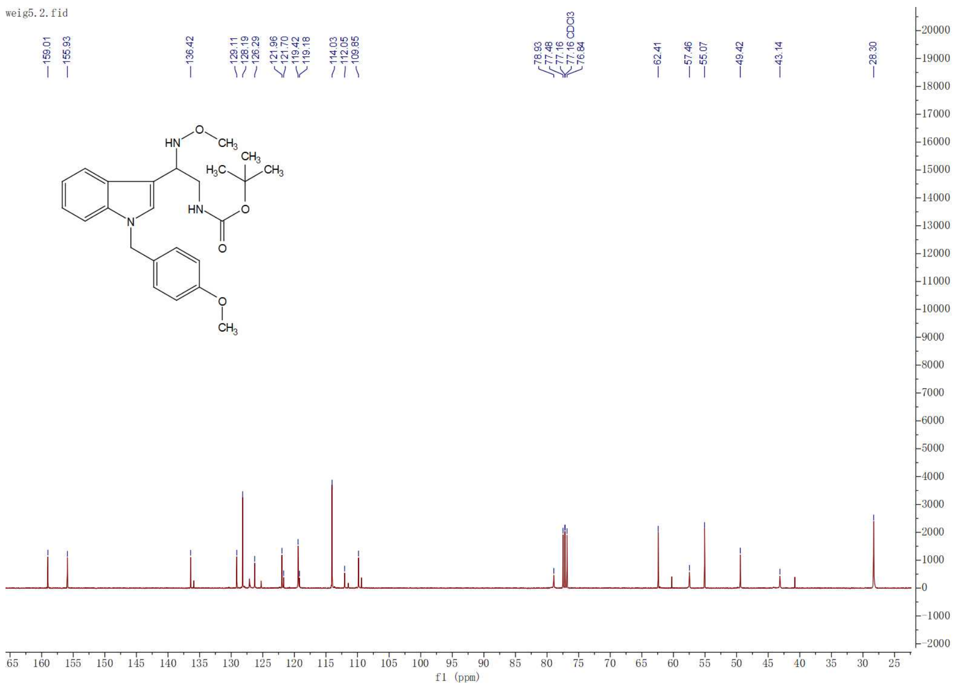

Figure S16. The ^{13}C NMR spectrum (400 MHz, chloroform-*d*) of **5**

6-bromo-1-(4-methoxybenzyl)-1H-indole (6)

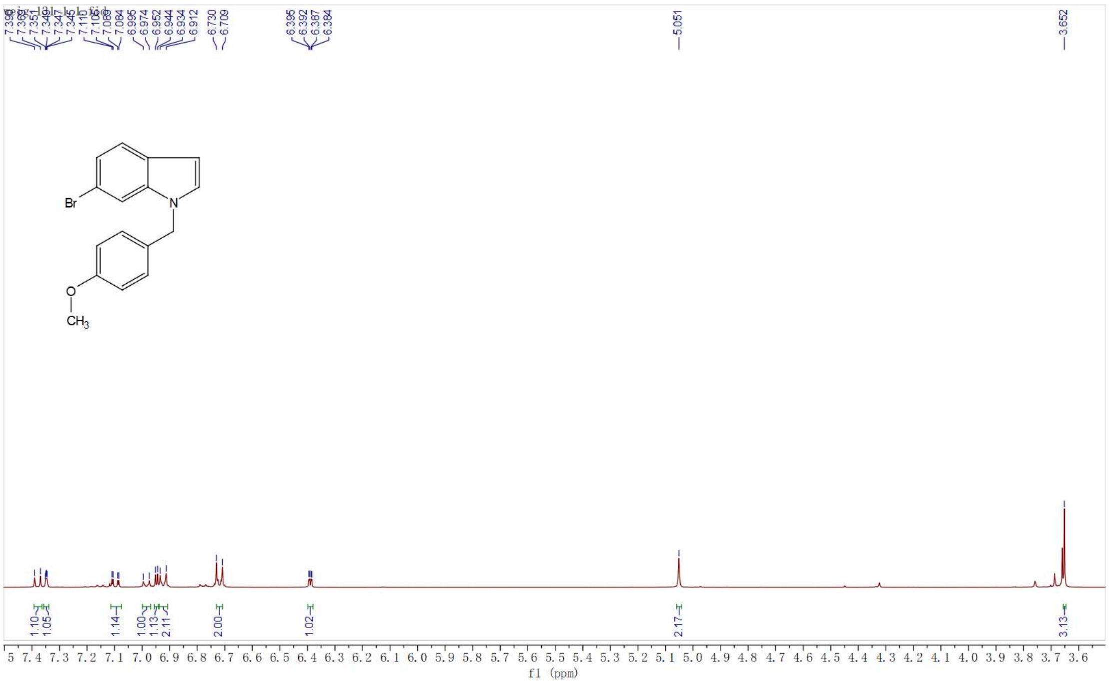

Figure S17. The ^{1}H NMR spectrum (400 MHz, chloroform-*d*) of **6**

methyl 3-(6-bromo-1-(4-methoxybenzyl)-1H-indol-3-yl)-3-oxopropanoate (7).

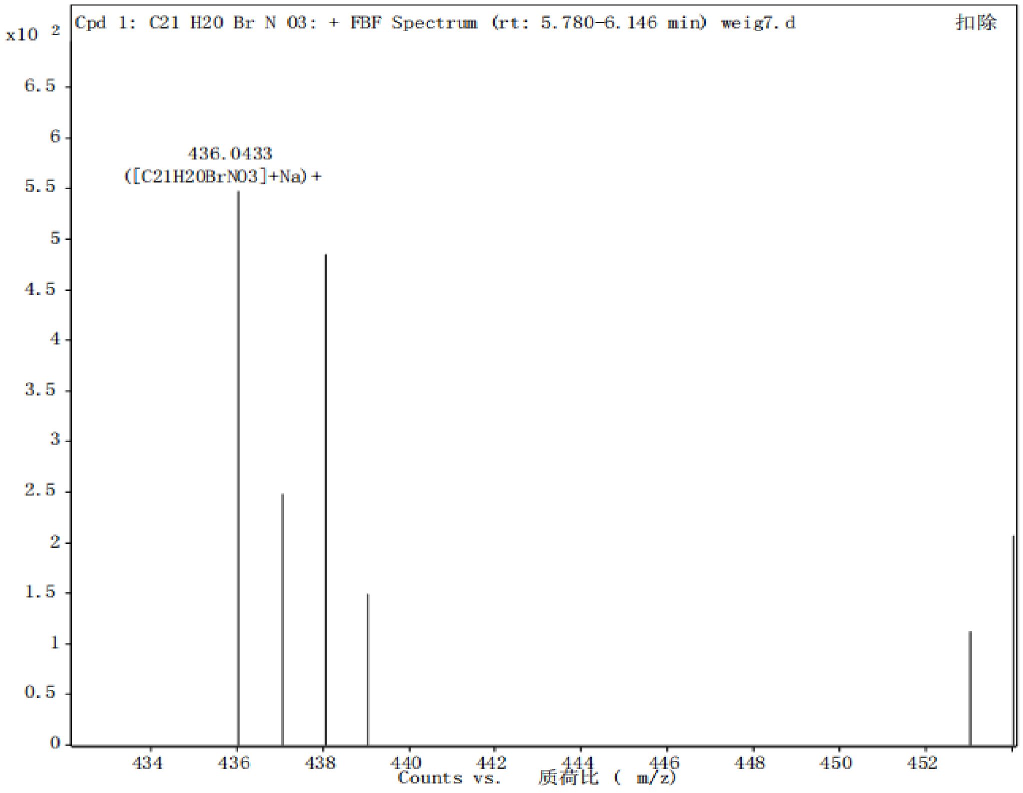

Figure S18. The HRESIMS spectrum of **7**

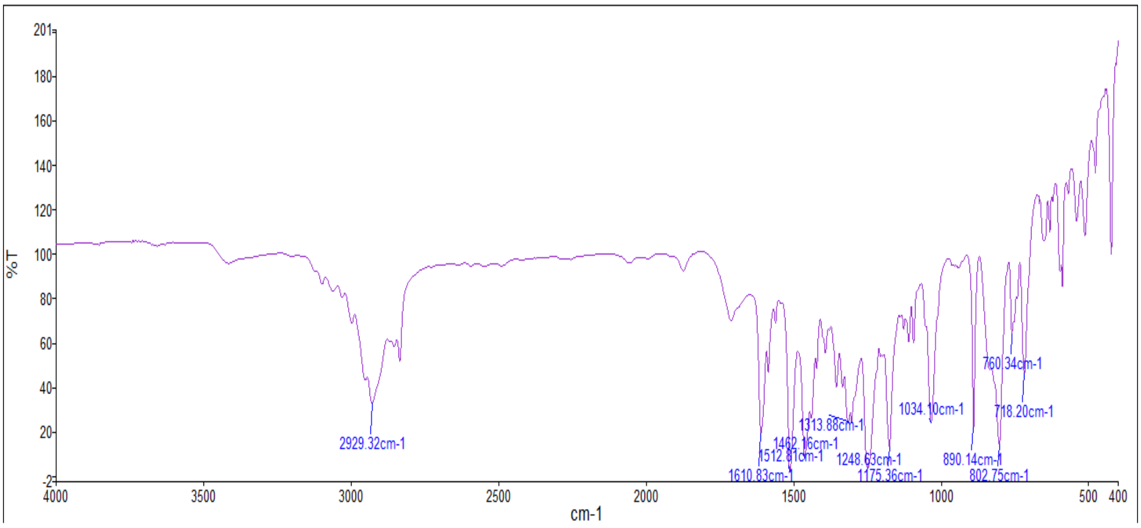

Figure S19. IR (KBr) spectrum of compound **7**

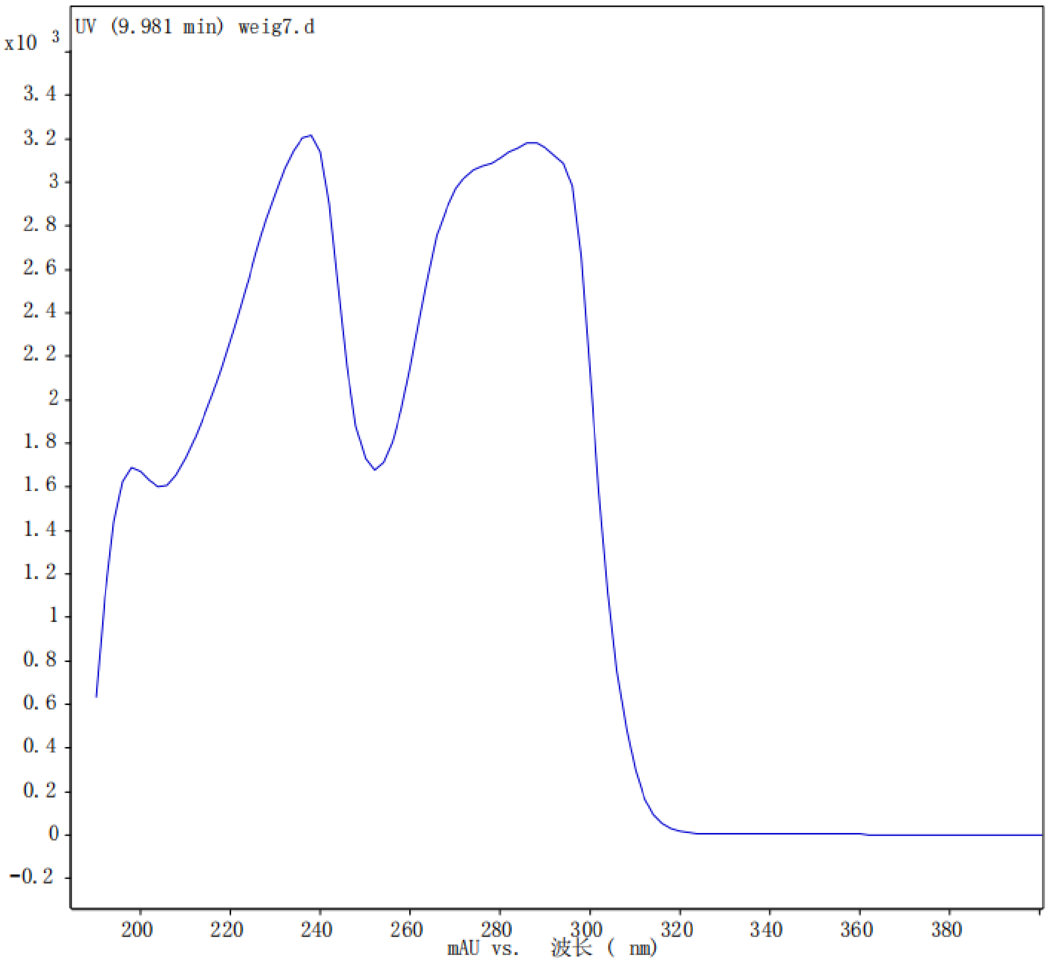

Figure S20. The UV spectrum of **7** (MeOH)

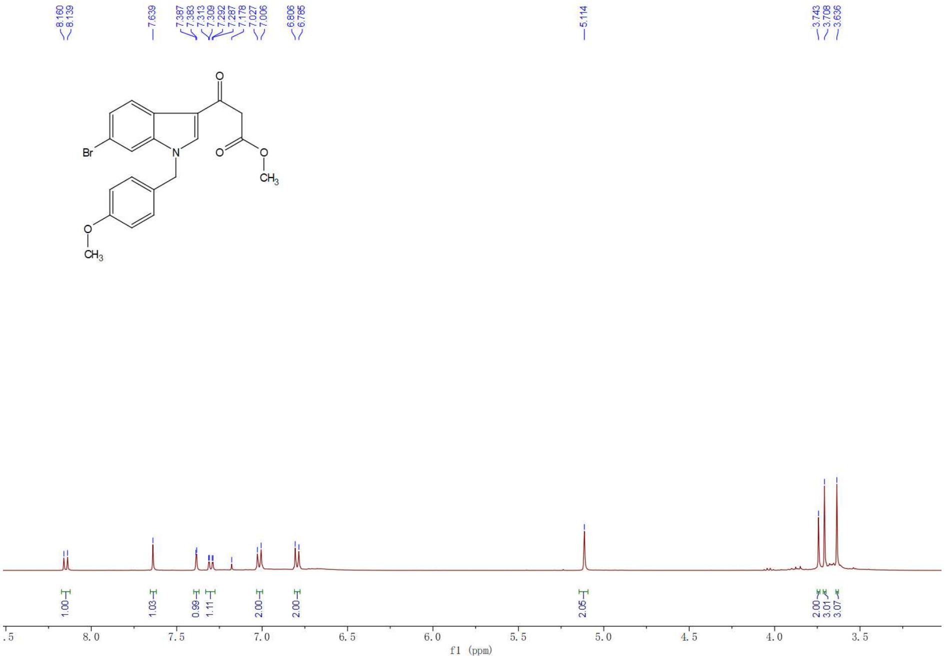

Figure S21. The ^{1}H NMR spectrum (400 MHz, chloroform-*d*) of **7**

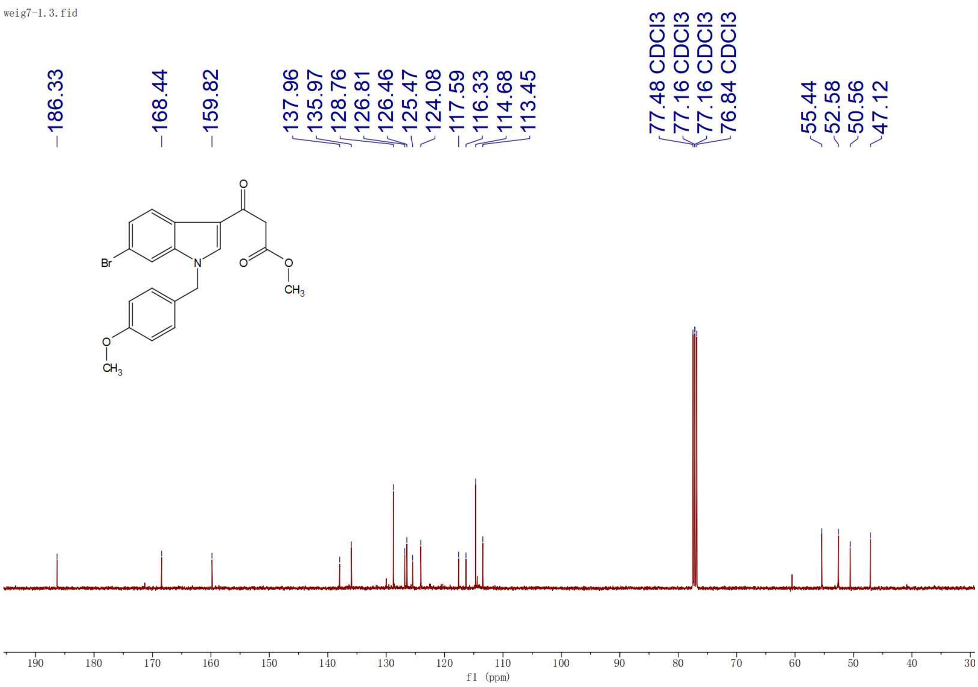

Figure S22. The ^{13}C NMR spectrum (400 MHz, chloroform-*d*) of **7**

methyl-3-(6-bromo-1-(4-methoxybenzyl)-1H-indol-3-yl)-2-diazo-3-oxopropanoate (8)

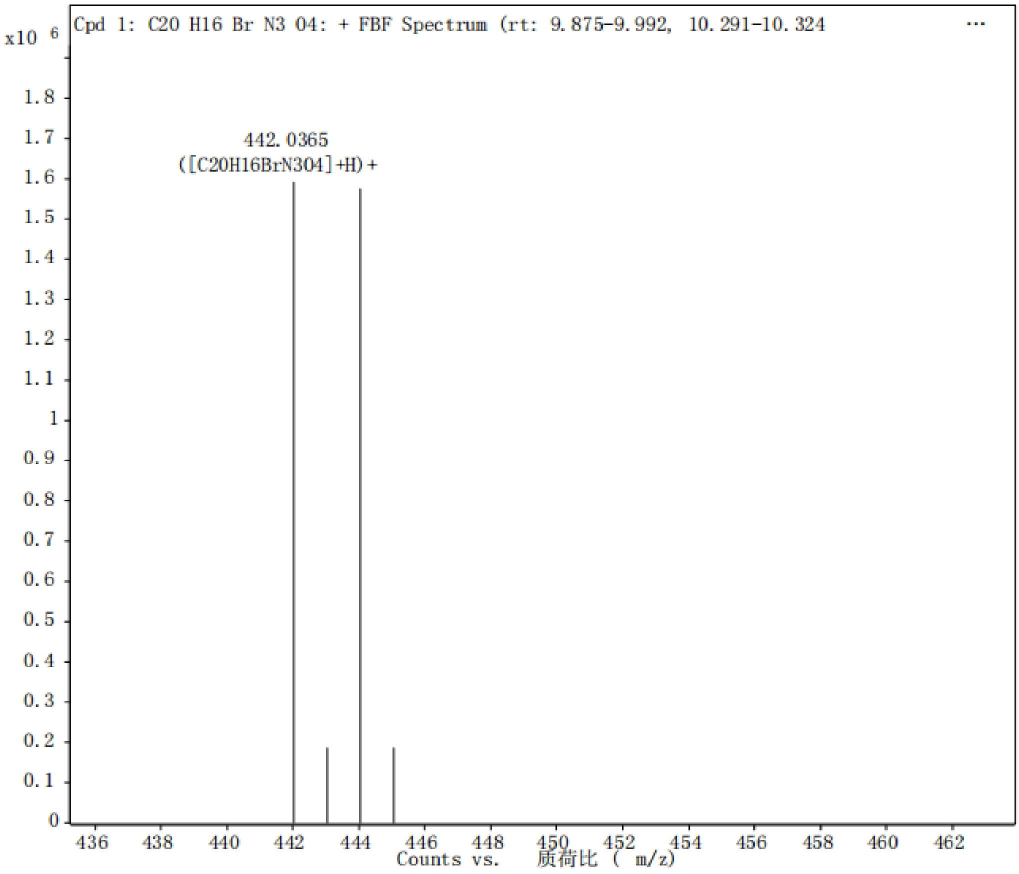

Figure S23. The HRESIMS spectrum of **8**

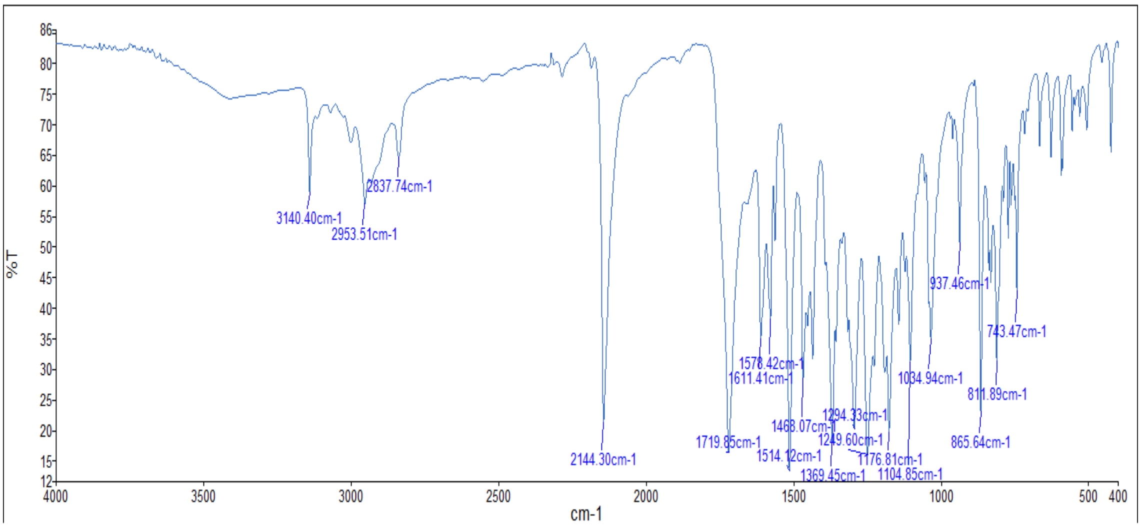

Figure S24. IR (KBr) Spectrum of compound **8**

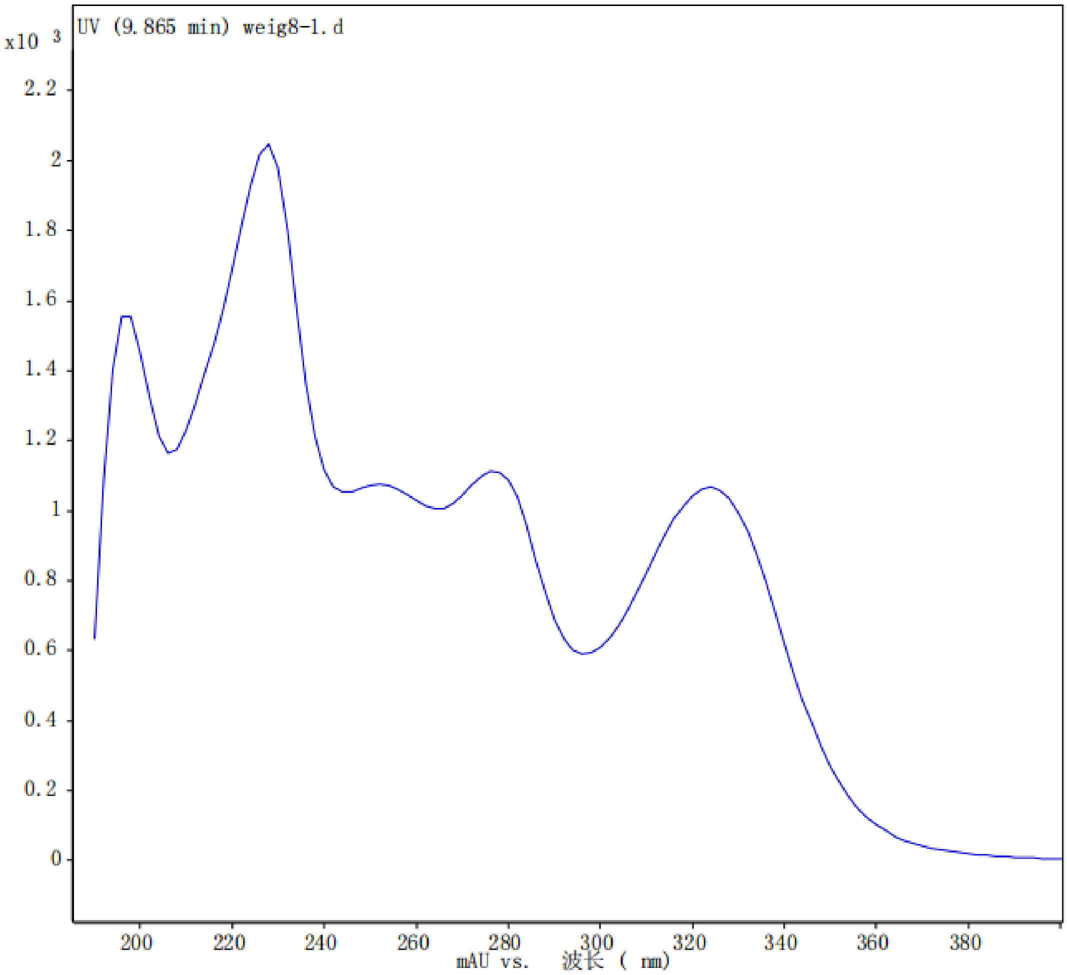

Figure S25. The UV spectrum of **8** (MeOH)

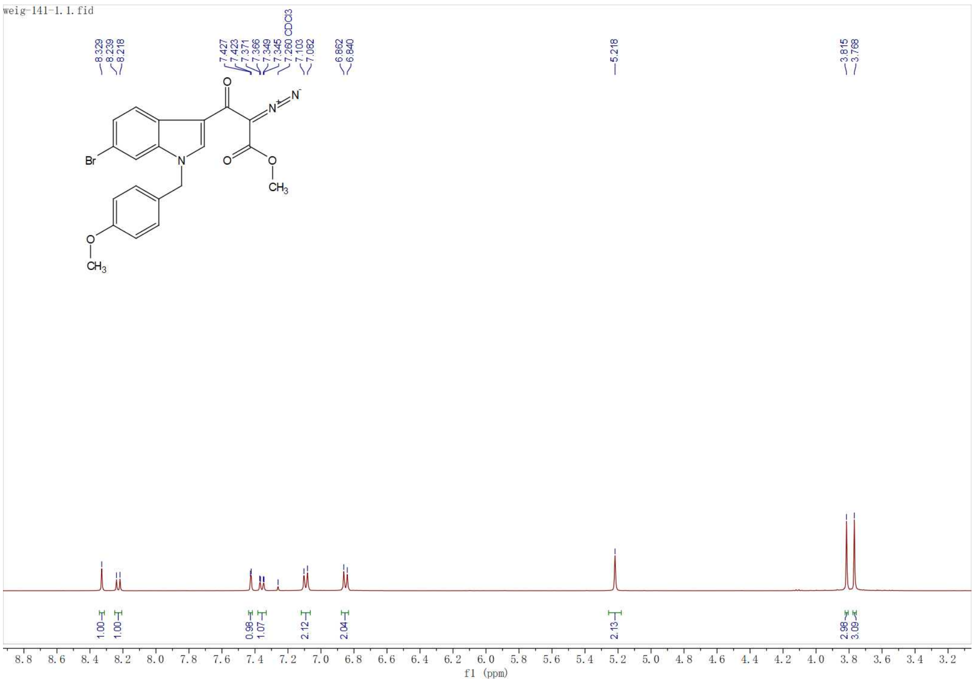

Figure S26. The ^{1}H NMR spectrum (400 MHz, chloroform-*d*) of **8**

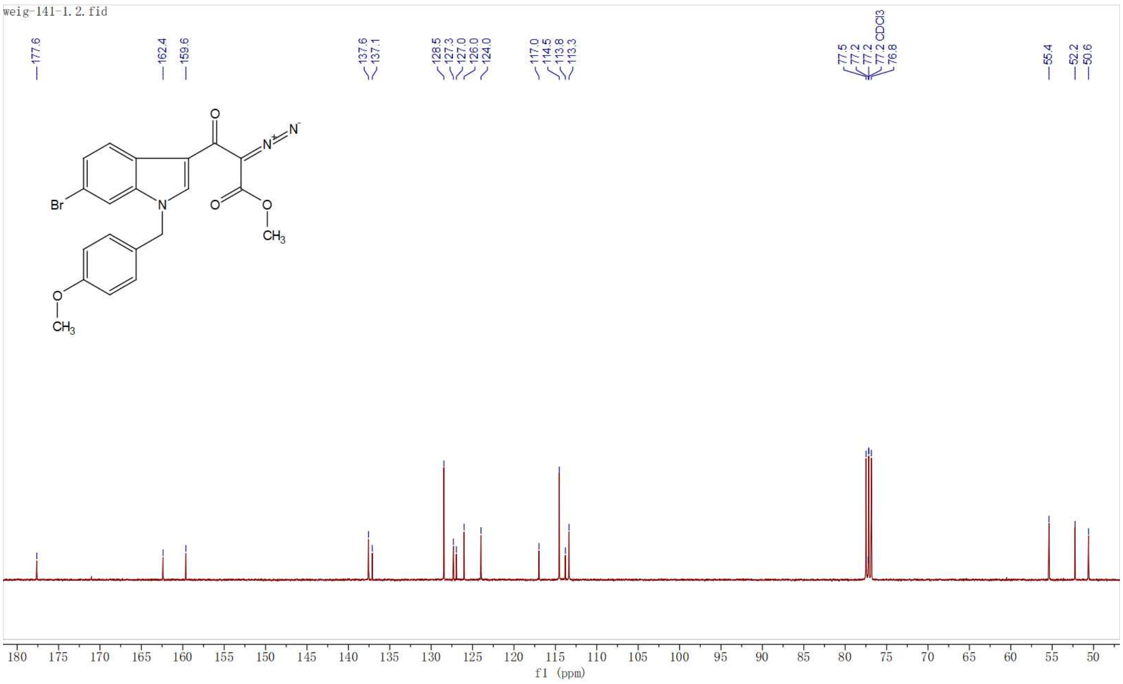

Figure S27. The ^{13}C NMR spectrum (400 MHz, chloroform-*d*) of **8**

Methyl-2-(6-bromo-1-(4-methoxybenzyl)-1H-indol-3-yl)-3-(methoxyamino)-3-oxo propanoate (9)

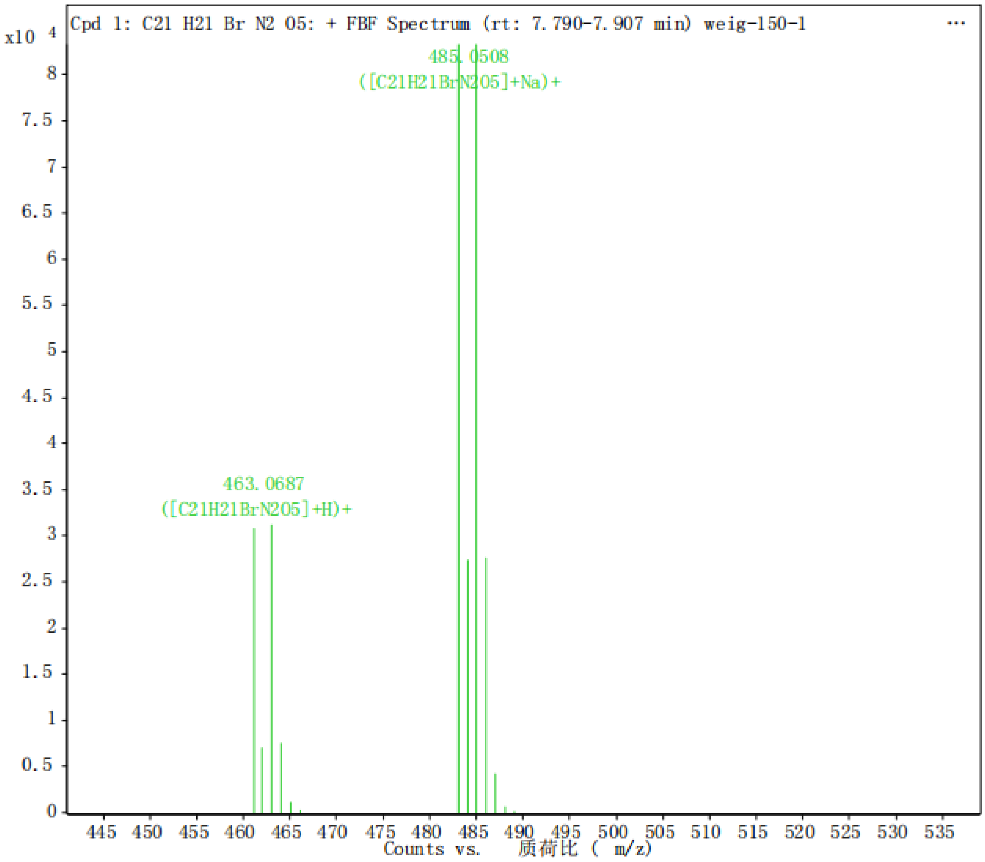

Figure S28. The HRESIMS spectrum of **9**

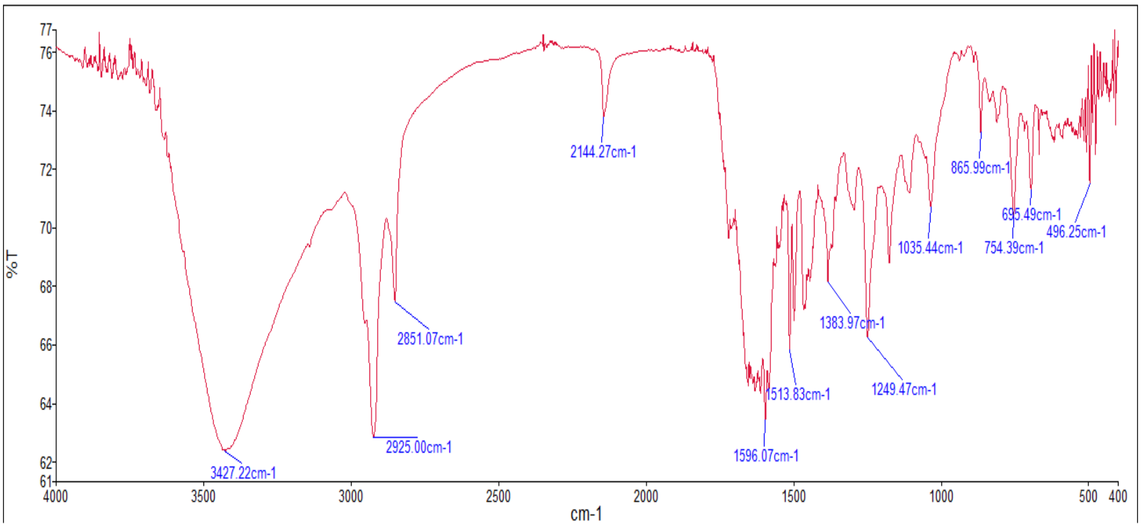

Figure S29. IR (KBr) spectrum of compound **9**

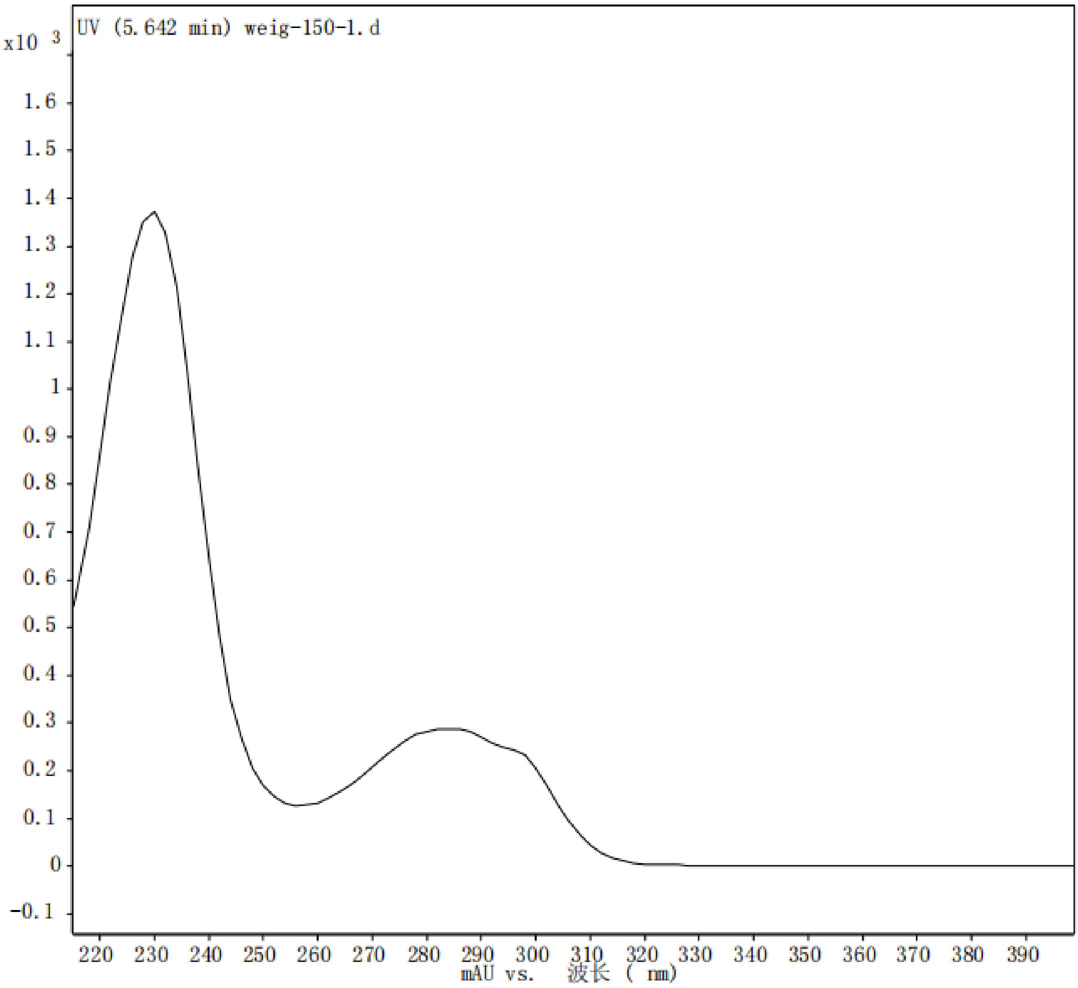

Figure S30. The UV spectrum of **9**

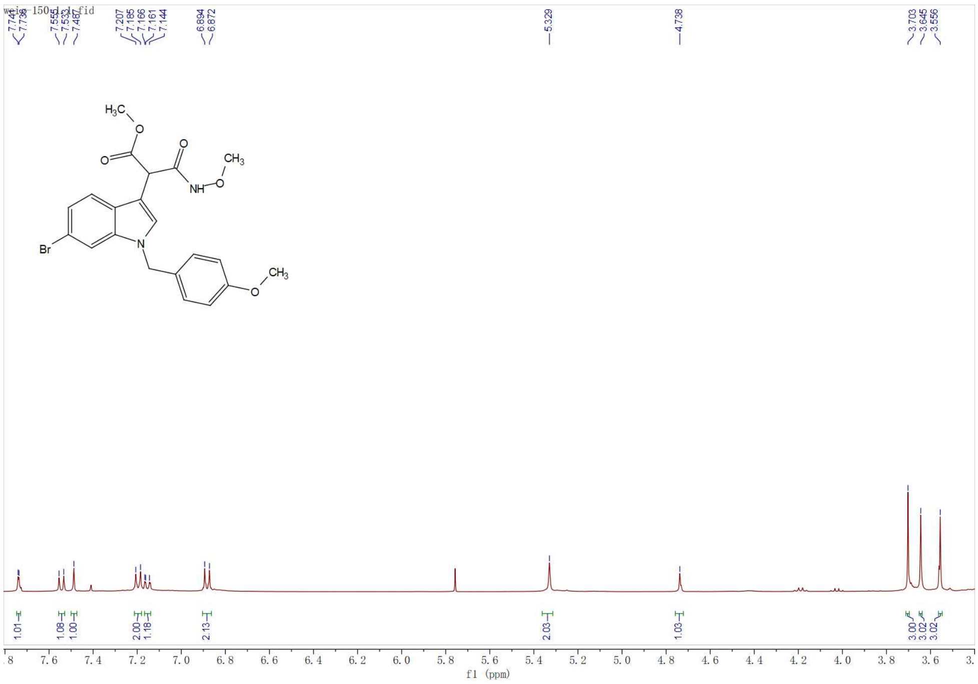

Figure S31. The ^{1}H NMR spectrum (400 MHz, DMSO-d_6) of **9**

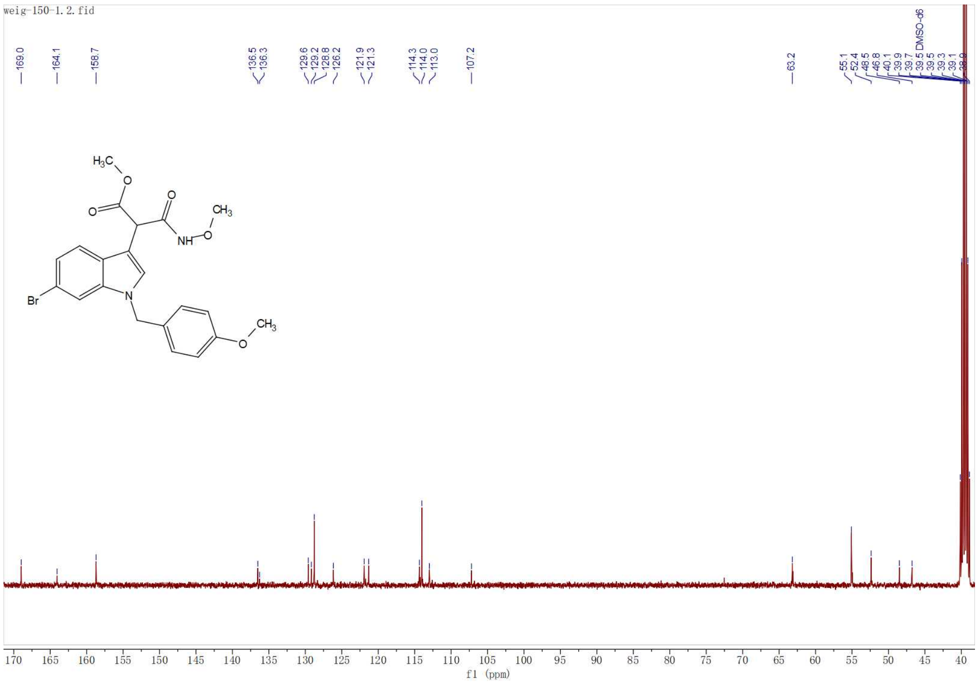

Figure S32. The ^{13}C NMR spectrum (400 MHz, DMSO-d_6) of **9**

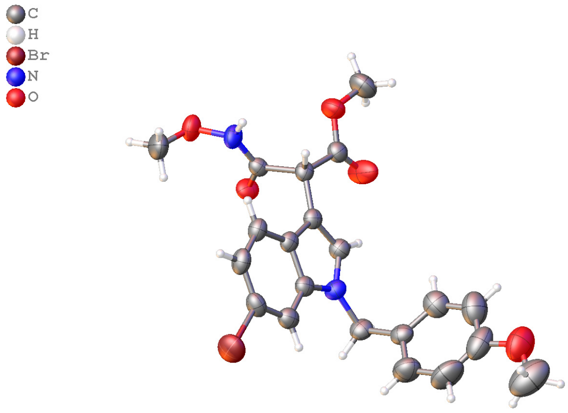

Figure S33. X-ray molecular structure of **9**

Table S1. The crystallographic data of **9**

Identification code	9
Empirical formula	$C_{21}H_{21}BrN_2O_5$
Formula weight	461.31
Temperature/K	295.01 (2)
Crystal system	orthorhombic
Space group	Pbca
a/Å	9.74420(10)
b/Å	8.63240(10)
c/Å	48.4549(6)
α/°	90
β/°	90
γ/°	90
VolμMe/Å^3	4075.82(4)
Z	8
ρ_{calc}g/cm^3	1.504
μ/mm^{-1}	3.056
F (000)	1888.0
Crystal size/mm^3	N/A × N/A × N/A
Radiation	Cu Kα (λ = 1.54184)
2Θ range for data collection/°	7.298 to 151.422
Index ranges	-12 ≤ h ≤ 12, -10 ≤ k ≤ 10, -60 ≤ l ≤ 21
Reflections collected	14792
Independent reflections	4074 [Rint = 0.0255, Rsigma = 0.0202]
Data/restraints/parameters	4074/0/265
Goodness-of-fit on F^2	1.139
Final R indexes [I>=2σ (I)]	R1 = 0.0699, wR2 = 0.1834
Final R indexes [all data]	R1 = 0.0790, wR2 = 0.1885
Largest diff. peak/hole / e Å^{-3}	0.80/-0.45
